# Modelling pancreatic β-cell inflammation in zebrafish identifies the natural product wedelolactone for human islet protection

**DOI:** 10.1242/dmm.036004

**Published:** 2019-01-23

**Authors:** Luis Fernando Delgadillo-Silva, Anastasia Tsakmaki, Nadeem Akhtar, Zara J. Franklin, Judith Konantz, Gavin A. Bewick, Nikolay Ninov

**Affiliations:** 1 Centre for Regenerative Therapies TU Dresden, Dresden 01307, Germany; 2 Paul Langerhans Institute Dresden of the Helmholtz Center Munich at the University Hospital Carl Gustav Carus of TU Dresden, German Center for Diabetes Reseach (DZD e.V.), Dresden 01307, Germany; 3 Diabetes Research Group, School of Life Course Sciences, Faculty of Life Sciences & Medicine, King's College London, London SE1 91UL, UK

**Keywords:** Diabetes, Inflammation, Insulin, Islet, Regeneration, Zebrafish, Beta-cells

## Abstract

Islet inflammation and cytokine production are implicated in pancreatic β-cell dysfunction and diabetes pathogenesis. However, we lack therapeutics to protect the insulin-producing β-cells from inflammatory damage. Closing this clinical gap requires the establishment of new disease models of islet inflammation to facilitate screening efforts aimed at identifying new protective agents. Here, we have developed a genetic model of Interleukin-1β (Il-1β)-driven islet inflammation in zebrafish, a vertebrate that allows for non-invasive imaging of β-cells and *in vivo* drug discovery. Live imaging of immune cells and β-cells in our model revealed dynamic migration, increased visitation and prolonged macrophage retention in the islet, together with robust activation of NF-κB signalling in β-cells. We find that Il-1β-mediated inflammation does not cause β-cell destruction but, rather, it impairs β-cell function and identity. *In vivo*, β-cells exhibit impaired glucose-stimulated calcium influx and reduced expression of genes involved in function and maturity. These defects are accompanied by α-cell expansion, glucose intolerance and hyperglycemia following a glucose challenge. Notably, we show that a medicinal plant derivative (wedelolactone) is capable of reducing the immune-cell infiltration while also ameliorating the hyperglycemic phenotype of our model. Importantly, these anti-diabetic properties in zebrafish are predictive of wedelolactone's efficacy in protecting rodent and human islets from cytokine-induced apoptosis. In summary, this new zebrafish model of diabetes opens a window to study the interactions between immune and β-cells *in vivo*, while also allowing the identification of therapeutic agents for protecting β-cells from inflammation.

## Abstract (precis)

**Summary:** A model of islet inflammation in zebrafish exhibits functional β-cell silencing and a lack of glucose responsiveness. A natural compound that rescues the glucose defect in zebrafish can protect human islets from inflammation.

## INTRODUCTION

Type 1 diabetes mellitus (T1DM) is an autoimmune reaction against the insulin-producing β-cells, which leads to cytokine-mediated β-cell loss. Type 2 diabetes mellitus (T2DM) is a pandemic metabolic disorder that affects 415-million people worldwide. T2DM often commences with insulin resistance triggered by over-nutrition and insufficient physical activity. In the long term, individuals with T2DM exhibit a loss of β-cell mass and function. Disease progression is potentiated by a reduced capacity of β-cells to undergo metabolic compensation, leading to additional β-cell stress and dysfunction due to hyperglycemia, a process culminating in a requirement for insulin replacement therapies (Kahn et al., 2014).

Current drug treatments targeting enhanced insulin secretion successfully ameliorate the symptoms of T2DM but are ineffective in triggering disease regression because they do not inhibit the pathways causing β-cell loss. An optimum treatment for T2DM should involve a component of disease modification as well as symptom management. Developing interventions that arrest or reverse β-cell loss and dysfunction has become an urgent priority (Marquard et al., 2015).

Inhibiting islet inflammation has emerged as an important objective for the protection of β-cells (Campbell-Thompson et al., 2016; Eizirik et al., 2009; Nordmann et al., 2017). It has been suggested that hyperglycemia in T2DM enhances the metabolic load on β-cells, causing an increase in the production of reactive oxygen species (ROS), in part, due to the oxidative nature of insulin protein folding and glucose metabolism (Ahmed Alfar et al., 2017). β-cell oxidative stress could promote inflammasome and caspase-1 activation, which can lead to the production and release of low levels of mature interleukin-1β (IL-1β). The production of IL-1β stimulates the release of several cytokines and chemokines that promote the migration and activation of macrophages, which are recruited to the islets and enhance the inflammatory environment further by exacerbating the release of cytokines (Donath et al., 2013).

IL-1β drives inflammation, in part, via activation of the nuclear factor-κB (NF-κB) signalling pathway (Lawrence, 2009). The experimental upregulation of NF-κB in β-cells using transgenic mice has been shown to cause reduced β-cell mass and diabetes (Salem et al., 2014), whereas genetic inhibition of NF-κB has protective effects (Eldor et al., 2006). Therefore, the identification of small molecules that protect β-cells from inflammation are likely to provide an effective strategy to prevent the failure of β-cells. Given the similarity between some of the inflammatory responses in T1DM and T2DM (Syed et al., 2002), β-cell protective agents could, in principle, be applicable as therapies for both types of diabetes.

Historically, drug discovery has been performed using biochemical or cell-based assays. Although these *in vitro* small-molecule screens can be conducted at high speed, they do not provide information about the efficacy of compounds *in vivo* in the natural endogenous environment. The low maintenance costs, rapid life cycle and high fecundity of zebrafish means that it offers a viable alternative for performing large-scale drug screens *in vivo* (Kamel and Ninov, 2017; Zon and Peterson, 2005). For example, the optical transparency of the developing zebrafish allows the observation of the pancreas non-invasively and over time. However, there are no zebrafish models of β-cell inflammation; such a model would allow the screening of compounds to identify β-cell protective agents.

To solve this problem, we developed a transgenic zebrafish model of β-cell inflammation. Since IL-1β is an important signal in the destruction of β-cells during an autoimmune attack in T1DM (Mandrup-Poulsen et al., 2010) and during β-cell dysfunction in T2DM (Dinarello et al., 2010), we used it to drive inflammation in our model. Expression of *il1b* in zebrafish β-cells led to activation of NF-κB signalling and macrophage infiltration into the islet. Live imaging of islets revealed that macrophages did not statically occupy the islet but instead underwent frequent and active migration in and out of the inflamed islet. Notably, β-cell mass was not reduced by *il1b* expression, but β-cell identity and function were impaired. For example, β-cells expressing *il1b* show impaired glucose-stimulated calcium influx. Notably, the natural product wedelolactone, which showed anti-inflammatory properties in our model, prevented hyperglycemia of zebrafish larvae in response to a glucose challenge and protected human β-cells from cytokine-induced damage. These data demonstrate the predictive power of our model for identifying translatable compounds that reduce islet inflammation and protect β-cells.

## RESULTS

### Expression of *il1b* leads to β-cell inflammation and immune-cell recruitment

**Fig. 1.**
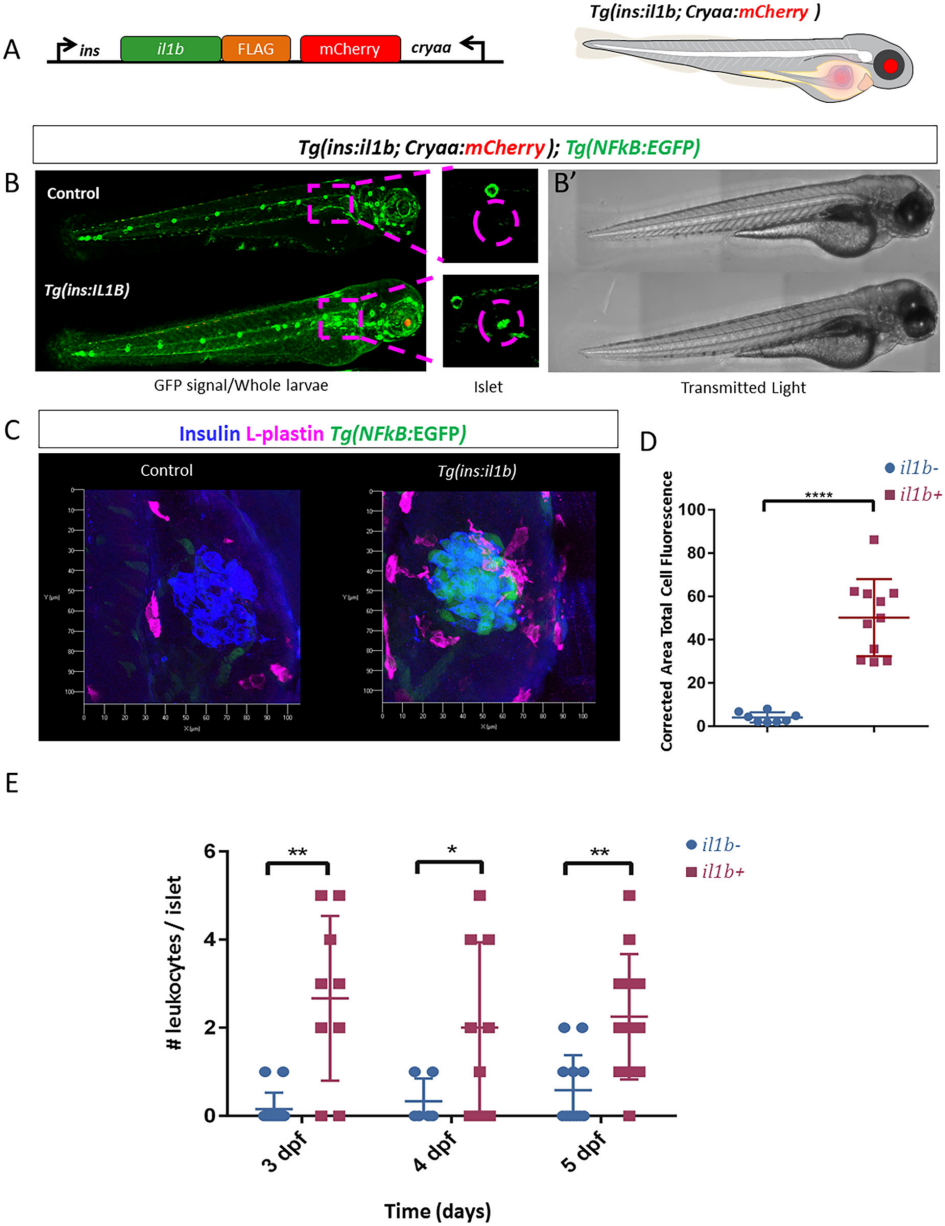
**Generation of a genetic model of chronic β-cell inflammation in zebrafish.** (A) Schematic representation of the genetic construct used to generate the transgenic model of chronic β-cell inflammation. The mature pro-inflammatory form of *il1b* was fused to the FLAG-peptide and cloned under the control of the insulin promoter. mCherry expression under the control of the crystalline (*cryaa*) promoter serves as a marker of transgenic animals (red eyes). (B) Representative confocal images (maximum projection) of *Tg(NF-kB:GFP)* larvae at 3 dpf in the presence or absence of *il1b* expression in β-cells. The top panel shows a control larva, whereas the bottom panel shows a *Tg(ins:il1b)* larva. The insets show high-magnification images of the islet region. There is strong GFP expression in the islets of *Tg(ins:il1b)* larvae compared to controls. Note that *Tg(ins:il1b)* larvae tend to exhibit higher GFP expression in the whole body compared to controls. (B′) Bright-field images of the larvae shown in B. Imaging in B was performed using tile-scan and the individual frames were automatically stitched together using the Tiles tool in the ZEN software (Zeiss) to render the entire larvae. (C) Representative confocal images of the primary islets from control and *Tg(ins:il1b)* larvae at 4 dpf in the transgenic background of a *Tg(NF-kB:GFP)* reporter (green). Immunostaining against insulin (blue) and L-plastin (magenta) marks the β-cells and the leukocytes, respectively. The islet from *Tg(ins:il1b)* larvae exhibits an increase in *NF-kB*:GFP expression compared to controls and the presence of leukocytes in the islet region. (D) Quantification of corrected area total cell fluorescence of *NF-kB*:GFP in the islets of control (blue) and *Tg(ins:il1b)* (red) larvae. Unpaired two-tailed *t*-test (Welch's correction), ****P<0.0001, mean±s.d. (E) Quantification of the number of leukocytes within the islet region of *Tg(ins:il1b)* (red) larvae compared to WT (blue) at 3, 4 and 5 dpf. Unpaired two-tailed *t*-test with Welch's correction, *P<0.05, **P<0.01, mean±s.d.

IL-1β is synthetized as an immature precursor and requires proteolytic cleavage by caspase-1 for its activation (Afonina et al., 2015). To cause β-cell inflammation, we designed a transgenic line expressing the presumptive mature form of zebrafish Il-1β under the control of the insulin promoter. To do this, we truncated the full-length Il-1β protein by removing the first 104 amino acids (out of 272), as Il-1β is cleaved at residue 104 by zebrafish Caspase A (Vojtech et al., 2012). For easy identification of transgenic animals, we introduced mCherry expressed under the retinal-specific promoter (*crystalline a*) (Fig. 1A).

To assess whether *Tg(ins:il1b)* animals exhibited β-cell inflammation, we analyzed the activity of NF-κB signalling using a transgenic reporter line, *Tg(NF-kB:GFP)*. This reporter expresses GFP under the control of NF-κB transcriptional responsive elements, such that GFP is expressed in a cell following the nuclear translocation and binding of an NF-κB dimer to the NF-κB-binding sites (Kanther et al., 2011). Whereas *NF-kB*:GFP activity was undetectable in the islets of wild-type (WT) 3 days post-fertilization (dpf) larvae, there was a robust activation of the reporter in *Tg(ins:il1b)* siblings (Fig. 1B,C)*.* We directly quantified GFP florescence intensity within the islets of WT and *Tg(ins:il1b)* larvae at 4 dpf, which confirmed a potent increase in GFP fluorescence (Fig. 1D).

An important sign of chronic inflammation is the recruitment of immune cells. To investigate whether immune cells were recruited to the islets in our model, we performed a time-course analysis from 3 to 5 dpf to quantify the numbers of islet-associated leukocytes in controls and *Tg(ins:il1b)* larvae. Using L-plastin as a leukocyte marker, we found that leukocytes were rarely associated with the islet in WT, whereas their numbers increased in *Tg(ins:il1b)* larvae (Fig. 1C,E; Movies 1 and 2)*.* To determine the activation status of the immune cells, we examined the expression of *tumour necrosis factor alpha* (*tnfa*) using a transcriptional GFP reporter line. Whereas we could not detect *tnfa* expression in the islet-associated leukocytes in WT larvae, approximately 40% of the cells were *tnfa*-positive in *Tg(ins:il1b)* at both 3 and 5 dpf (Fig. S1). Thus, the expression of Il-1β in β-cells leads to the recruitment of intra-islet immune cells, a hallmark of inflammation.

### Live imaging reveals dynamic interactions between immune cells and β-cells

**Fig. 2.**
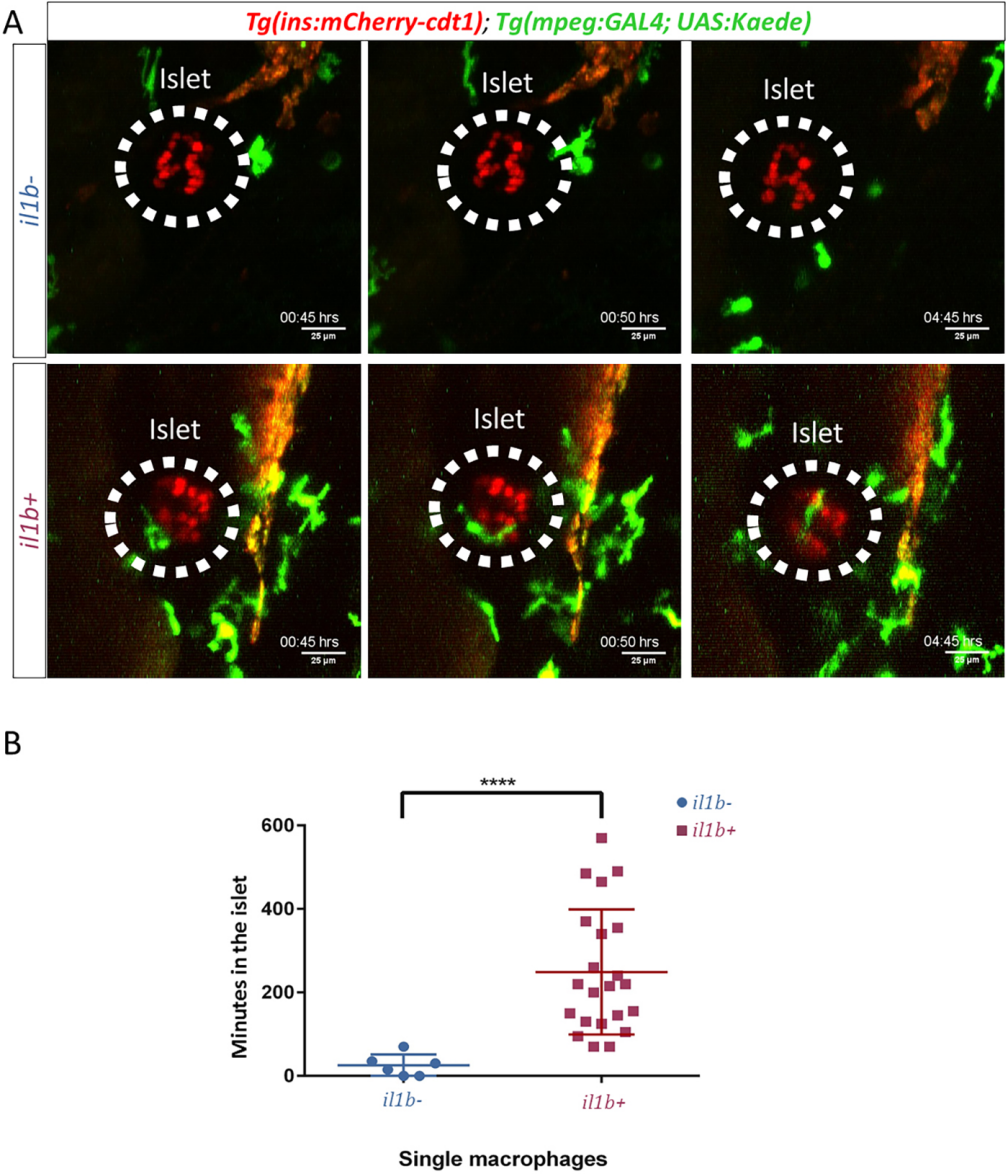
**Time-lapse imaging reveals dynamic interactions between β-cells and macrophages under chronic inflammation.** (A) Representative snapshots from time-lapse movies from control and *Tg(ins:il1b)* larvae. *Tg(ins:mCherry)* labels the β-cells (red), whereas *Tg(mpeg1:GAL4);Tg(UAS-Kaede)* labels the macrophages (green). β-cells and macrophages were imaged every 5 min for 10 h starting at 4.5 dpf. The elapsed time in hours (hrs) is indicated. (B) Plot showing the time that individual macrophages spend in the islet region as defined using a region of interest (ROI) (*n*=5 animals each for *il1b*− and *il1b*+). In controls (blue), the macrophages rarely visit the islet. In *Tg(ins:il1b)* larvae (red), the macrophages show an increase in the time they spend in the islet region. Unpaired two-tailed *t*-test with Welch's correction, ****P<0.0001, mean±s.d.

To understand the dynamics of the interaction between immune cells and β-cells *in vivo*, we performed confocal time-lapse imaging of macrophages in living larvae at 4.5 dpf. We used *Tg(mpeg1:GAL4); Tg(UAS:Kaede)* to specifically label macrophages with the GFP Kaede, whereas β-cells were labelled with nuclear mCherry; this allowed the two populations to be easily distinguished (Fig. 2). Live imaging showed that macrophages rarely visited WT islets and did not persist in proximity to the β-cells (Fig. 2A; Movie 3). In contrast, there was an increase in the frequency of macrophage visitation to the islet region in *Tg(ins:il1b)* larvae (Fig. 2A; Movie 4). These macrophages exhibited frequent shape changes and migrated in and out of the islet. Overall, there was an increase in the frequency of macrophage migration into the islet. Moreover, on average, the macrophages spent longer in the islets of *Tg(ins:il1b)* larvae compared to controls (Fig. 2B). In summary, the increase in the number of leukocytes we observed in our fixed-sample experiments was the result of both more frequent visitations by macrophages and increased interactions with the β-cells.

### Transcriptional analysis reveals impaired β-cell identity and downregulation of genes involved in β-cell function

**Fig. 3.**
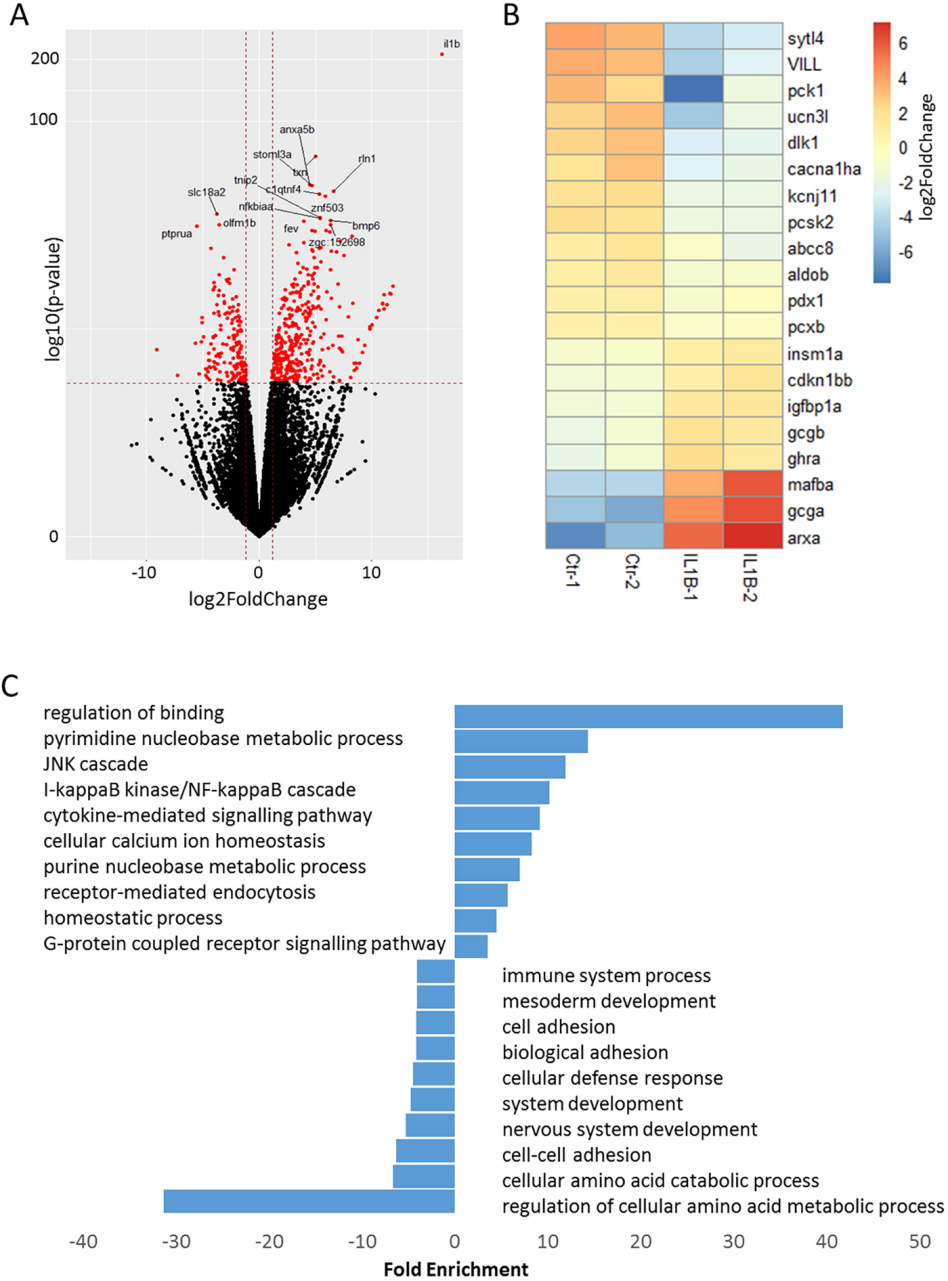
**Transcriptomic analysis of β-cells from *Tg(ins:il1b)* animals reveals changes in genes associated with β-cell identity and function.** (A) Volcano plot displaying the distribution of gene expression in β-cells from *Tg(ins:il1b)* at 3 months post-fertilization. The *y*-axis shows the log_10_ *P*-value. The *x*-axis shows the fold change in gene expression. The perpendicular dashed lines to the *x*-axis marks genes with ±1.5 log_2_ fold change in expression (to the left and right of the lines, respectively). The parallel line to the *x*-axis marks genes differentially expressed with a *P*<0.05 and a false discovery rate (FDR) <0.05 (red dots). We found a total of 1245 differentially expressed genes in β-cells from *Tg(ins:il1b)* animals. (B) Heatmap of selected genes associated with β-cell function and identity. In *Tg(ins:il1b)*-expressing β-cells, genes associated with β-cell function and identity, such as *cacna1ha*, *ucn3l* and *sytl4*, were downregulated, whereas genes normally expressed in α-cells, such as *gcga*, *mafba* and *arxa*, were upregulated. (C) Graph showing the top ten most enriched Gene Ontology (GO) terms based on the top 100 most upregulated or downregulated genes. The *x*-axis shows the fold enrichment from the Panther GO, with a *P*-value less than 0.05.

To investigate the specific effects that chronic inflammation has on the β-cell transcriptome, we conducted mRNA-sequencing, gene-enrichment and network analysis in β-cells isolated from *Tg(ins:il1b)* animals and WT siblings. To collect enough cells for transcriptomic analysis, β-cells were sorted by fluorescence-activated cell sorting (FACS) (Fig. S2) from fish aged 3 months post-fertilization (mpf), which were viable and healthy. We identified 1245 differentially expressed genes with a false discovery rate (FDR) <0.05 (Fig. 3A). Gene Ontology (GO) analysis of the top 100 significantly upregulated genes in inflamed β-cells showed an increase in genes related to the NF-κB and the JNK cascades, cytokine signalling and cellular calcium ion homeostasis (Fig. 3B). For example, genes associated with inflammation, such as *TNF receptor-associated factor 3* (*traf3*), *cxcl18b* and *nuclear factor of kappa light polypeptide gene enhancer in B-cells inhibitor, epsilon* (*nfkbie*), were strongly upregulated. In agreement with this inflammatory signature, there was a persistent increase in leukocyte infiltration in the islets of *Tg(ins:il1b)* animals compared to WT siblings when examined at a later stage (30 dpf) (Fig. S3). On the other hand, the top 100 downregulated genes showed a strong relation to amino-acid metabolism, cellular adhesion and cellular defence response (Fig. 3B).

We further performed a hierarchical clustering by Pearson correlation to trace genes involved in β-cell function and identity. Important regulators of β-cell maturity and function were expressed at lower levels in β-cells from *Tg(ins:il1b)* larvae compared to controls (Fig. 3C). For example, *urocortin 3l* (*ucn3l*), *pancreatic and duodenal homeobox 1* (*pdx1*), *cacna1ha*, *sytl4* and the T2DM risk gene *kcnj11*, which encodes the components of the ATP-sensitive potassium (K-ATP) channel, were downregulated in the *il1b*-expressing β-cells. Notably, genes involved in specification of other hormonal pancreatic cells, such as α-cells, were upregulated. For example, there was increased expression of *arxa*, *mafba*, *gcga* and *gcgb*, genes that should be restricted to α-cells (Fig. 3C). This analysis shows a deregulation of hormonal expression, especially glucagon, and points to a possible impairment of β-cell identity and function under chronic inflammation.

### Chronic inflammation impairs the glucose-stimulated calcium influx of β-cells *in vivo*

**Fig. 4.**
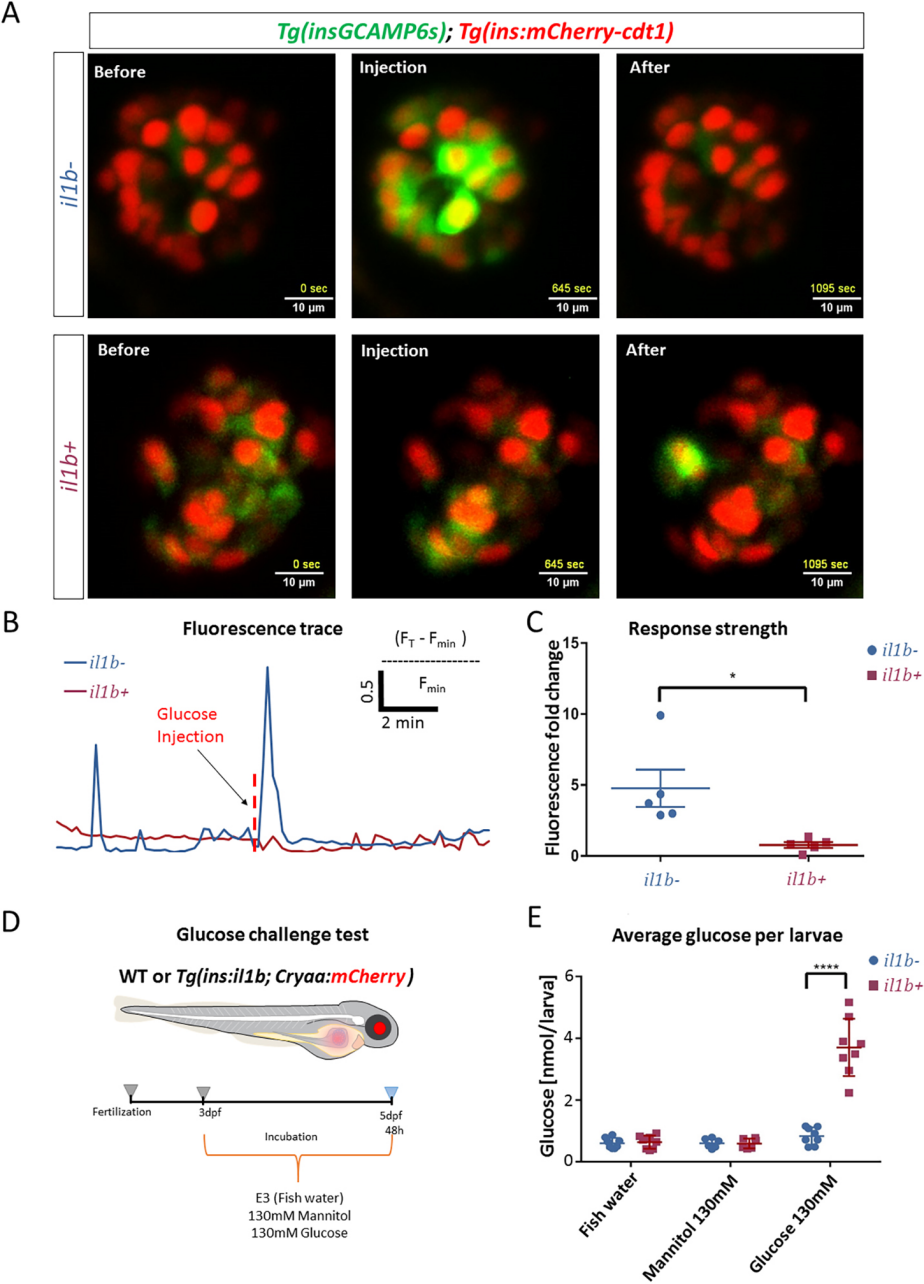
**β-cell inflammation leads to reduced glucose responsiveness of β-cells and hyperglycemia following a glucose challenge.** (A) Snapshots from live imaging of 4.5 dpf larvae expressing GCaMP6s and nuclear mCherry in the β-cells. The images show the GCaMP6s fluorescence before, during and after glucose stimulation. The larvae were mounted and imaged using a confocal microscope. The β-cells were stimulated with 12.5 mM glucose solution by intracardiac injection. (B) Traces of GCaMP6s fluorescence intensity over time for the islets shown in A. The islet of the WT larva showed a strong and coordinated increase in GCaMP6s fluorescence in response to glucose injection, indicating glucose-stimulated calcium influx, whereas the islet of the *Tg(ins:il1b)* larva did not respond to glucose. (C) Graph showing the GCaMP6s fluorescence-intensity fold change before and after the glucose injection. *Tg(ins:il1b)* β-cells showed a drastically reduced glucose-stimulated calcium influx compared to controls. Unpaired two-tailed *t*-test with Welch's correction, *P<0.05. *n*=5 larvae, mean±s.d. (D) A model showing the design of a glucose challenge in zebrafish larvae. The glucose challenge consists of incubating the larvae in three solutions: fish water (E3), 130 mM mannitol as an osmotic control, and 130 mM glucose. The larvae were incubated at 3 dpf and the glucose was measured at 5 dpf. (E) Plot showing glucose concentration following a glucose challenge in controls and *Tg(ins:il1b)* larvae. The *Tg(ins:il1b)* larvae show similar free glucose levels as WT larvae in fish water (E3) and mannitol. Following glucose challenge, the *Tg(ins:il1b)* larvae show an approximately 3.5-fold increase in free glucose compared to WT siblings. Two-way ANOVA with Sidak's multiple-comparison test; ****P<0.0001; each data point represents a pool of ten larvae; mean±s.d.

To test whether chronic inflammation impairs β-cell function *in vivo*, we performed live imaging of glucose-stimulated calcium influx using a β-cell-specific GCaMP6s reporter. Our laboratory previously developed this model to measure glucose responsiveness of zebrafish β-cells (Singh et al., 2017). Using the GCaMP6s system, we first characterized glucose-induced calcium influx in 4.5 dpf WT larvae mounted in agarose. Stimulation with 12.5 mM glucose via injection in the circulation produced an immediate β-cell response, with an average 4.7-fold increase in normalized GCaMP6s intensity (Fig. 4A,C; Movie 5). In contrast, the *il1b*-expressing β-cells showed a diminished response to glucose stimulation (Fig. 4A,C; Movie 6). Interestingly, we observed frequent endogenous calcium spikes in β-cells of WT larvae in the absence of glucose stimulation (Movies 5 and 6). This endogenous β-cell activity is likely a response to the basal circulating glucose levels in larvae (V. Salem, L.F.D.-S. and K. Suba et al., unpublished). The expression of *il1b* did not appear to change the endogenous calcium spikes in β-cells (Movie 5). This result illustrates that chronic inflammation does not interfere with the ability of the β-cells to respond to basal glucose levels but specifically inhibits the responsiveness to increased glucose concentrations.

### β-cell inflammation and metabolic stress lead to hyperglycemia

Since our results indicated an impaired glucose response of β-cells, we challenged the WT and *Tg(ins:il1b)* larvae with glucose to assess their ability to regulate sugar levels. Larvae were incubated in normal fish water or fish water supplemented with glucose from 3 to 5 dpf (130 mM). To account for any potential confounding effects of hyperosmolarity, we incubated the larvae in mannitol (130 mM) as an additional control. After 2 days of the challenge, we measured free glucose levels in the larvae (Fig. 4D). Under normal conditions, chronic inflammation did not change significantly the free glucose concentrations. Conversely, in response to glucose incubation, the *Tg(ins:il1b)* larvae showed significantly elevated glucose compared to WT siblings (Fig. 4E). To assess glucose tolerance as previously described (Matsuda et al., 2017), we incubated 4.8 dpf larvae in glucose for 60 min, washed the glucose and measured whole-animal glucose levels at 1, 30 and 60 min (Fig. S4A). *Tg(ins:il1b)* larvae showed glucose intolerance compared to WT (Fig. S4B), consistent with the defect in glucose responsiveness we documented using calcium imaging.

### Chronic inflammation is associated with α- and β-cell expansion

**Fig. 5.**
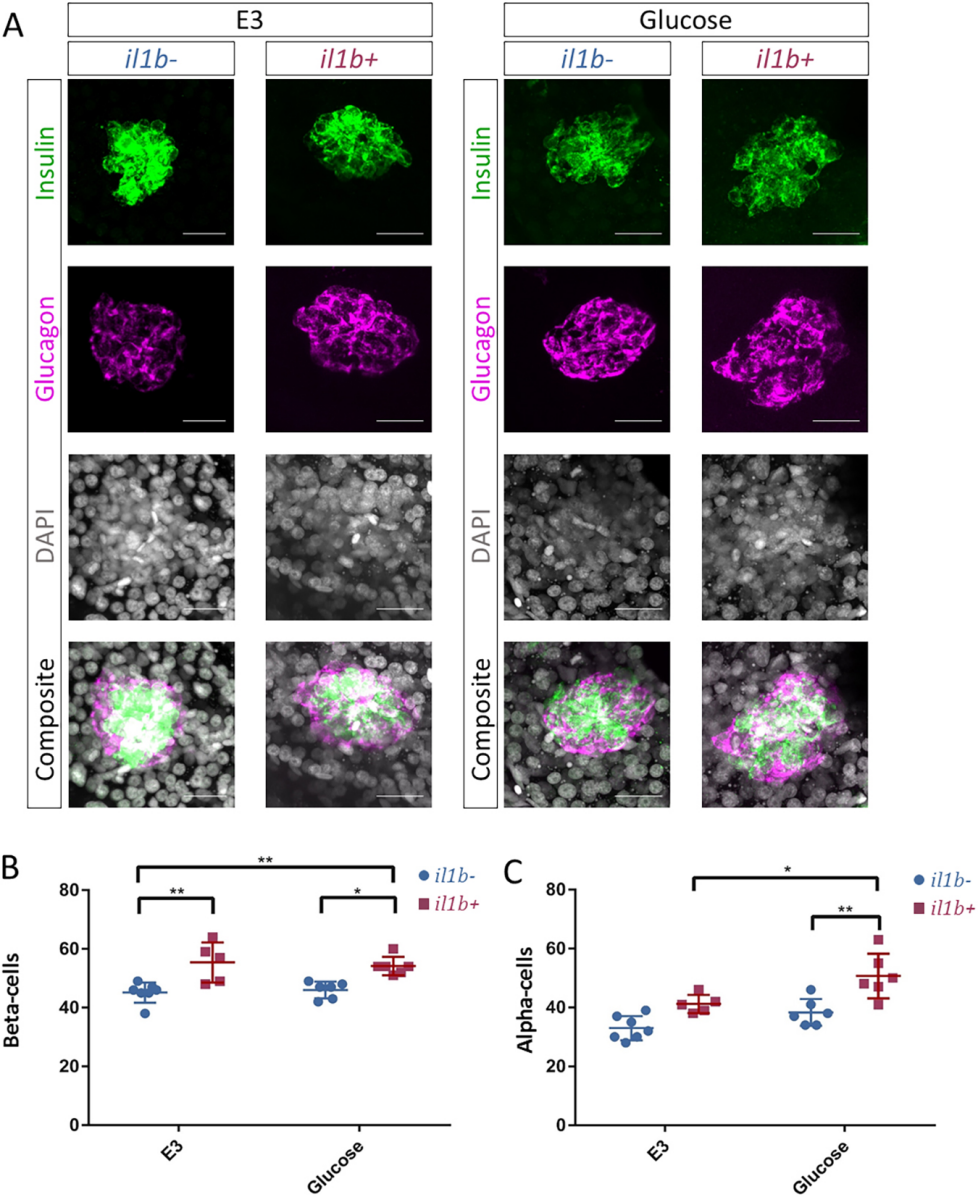
**Chronic inflammation and high-glucose exposure leads to α-cell expansion.** (A) Confocal images (maximum projections) of islets from WT and *Tg(ins:il1b)* larvae at 5 dpf following incubation in either E3 or 130 mM glucose from 3 to 5 dpf. Immunostainings against insulin (green) and glucagon (magenta) mark the β-cells and the α-cells, respectively. (B) Quantification of the number of β-cells in WT and *Tg(ins:il1b)* larvae. *Tg(ins:il1b)* larvae exhibit an increase in β-cells in both E3 and glucose. Two-way ANOVA with Sidak's multiple-comparison test; *P≤0.05, **P≤0.01. Mean±s.d. (C) Quantification of the number of α-cells in WT and *Tg(ins:il1b)* larvae. The number of α-cells did not differ significantly (*P*<0.5) between WT and *Tg(ins:il1b)* in E3. However, there were significantly more α-cells in *Tg(ins:il1b)* larvae compared to WT in the glucose-treated group. Two-way ANOVA with Sidak's multiple-comparison test; *P≤0.05, **P≤0.01. Scale bars: 20 μm.

To determine whether the defect in glucose regulation might be a consequence of reduced β-cell mass, we quantified the number of insulin-positive cells in the *Tg(ins:il1b)* larvae under normal conditions and following a glucose challenge from 3 to 5 dpf. We found that *Tg(ins:il1b)* larvae possess higher numbers of insulin-positive cells compared to controls both under basal conditions and in the glucose challenge (Fig. 5A,B). Thus, the defect in glucose regulation that we observed in larvae is likely not a result of a reduction in β-cell numbers but involves changes in β-cell function, as indicated by our analysis of glucose-stimulated calcium influx.

We also studied the number of glucagon-positive cells. We found that α-cell numbers were elevated in the glucose-treated *Tg(ins:il1b)* larvae but not under basal conditions (Fig. 5A,C), suggesting that the combination of chronic inflammation and higher glucose levels can lead to α-cell expansion. Interestingly, in juvenile fish (30 dpf), at which point the animal regulates natural postprandial surges in blood sugar, *Tg(ins:il1b)* animals presented an increase in the α-cell population (Fig. S5A-D). Moreover, there was also an increase in the proportion of glucagon and insulin bi-hormonal cells in *Tg(ins:il1b)* fish (Fig. S5E), recapitulating the defects in hormonal expression suggested by our RNA-sequencing experiment.

### Wedelolactone ameliorates the hyperglycemia in *Tg(ins:il1b)* larvae

**Fig. 6.**
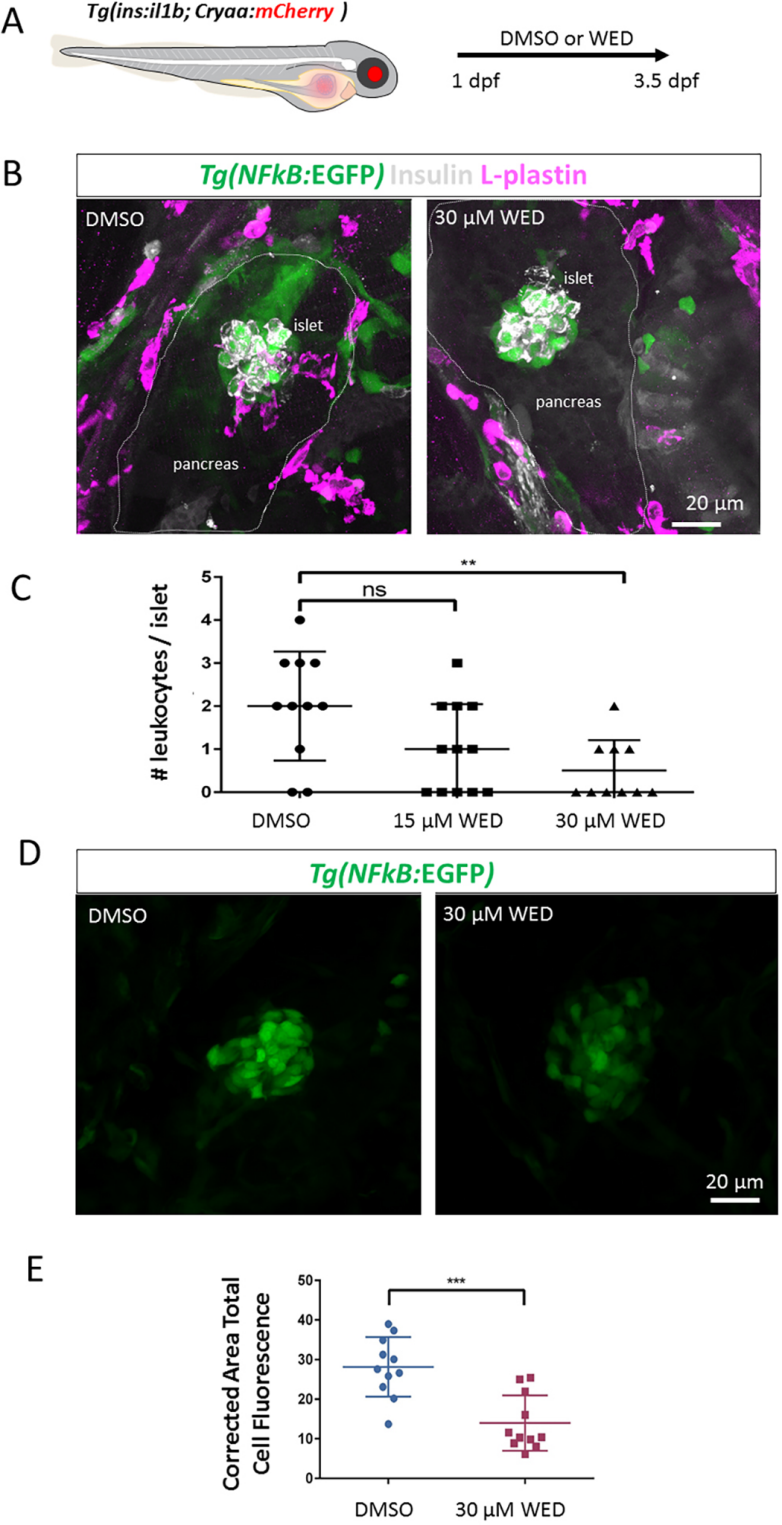
**Wedelolactone treatment ameliorates immune-cell infiltration and reduces islet NF-κB signalling activation.** (A) Schematic of the experimental approach. *Tg(ins:il1b);Tg(NF-kB:GFP)* embryos were treated with DMSO or wedelolactone (WED) from 1 to 3.5 dpf. At 3.5 dpf, the presence of L-plastin-positive leukocytes in the islet region was quantified. (B) Representative confocal images of *Tg(ins:il1b);Tg(NF-kB:GFP)* embryos treated with DMSO or 30 μM wedelolactone. Whereas the leukocytes have infiltrated the islet in DMSO controls, there is a reduction in islet-associated immune cells following wedelolactone treatment. (C) Quantification of the number of L-plastin-expressing cells in the islets of DMSO- and wedelolactone-treated larvae. One-way ANOVA with Sidak's multiple-comparison test; **P≤0.01; ns, not significant. Mean±s.d. (D) Representative confocal images of the islets of *Tg(ins:il1b);Tg(NF-kB:GFP)* embryos treated with DMSO or wedelolactone from 1 to 4 dpf. Wedelolactone-treated larvae show a visible reduction in *NF-kB:*GFP fluorescence intensity in the islet region at 4 dpf. (E) Quantification of corrected area total cell *NF-kB:*GFP fluorescence in the islet region following DMSO or wedelolactone treatment, showing a reduction in *NF-kB:*GFP fluorescence. Unpaired two-tailed *t*-test with Welch's correction, ***P≤0.05. Mean±s.d.

Our goal was to develop a β-cell inflammation model for drug testing. As a proof-of-principle, we investigated whether anti-inflammatory agents could help ameliorate the defect in glucose regulation in *Tg(ins:il1b)* larvae. To this end, we focused on the natural product wedelolactone, which is traditionally used in Chinese medicine to treat inflammatory conditions. Wedelolactone has also been shown to inhibit the NF-κB signalling pathway, and to act as a potent and selective inhibitor of 5-lipoxygenases (5-LOX), which converts arachidonic acid into leukotrienes (Sarveswaran et al., 2012). First, we tested whether wedelolactone exhibits anti-inflammatory properties in our model. We treated embryos with DMSO or wedelolactone from 1 to 3 dpf and counted the number of immune cells within the islet (Fig. 6A,B). Importantly, whereas DMSO-treated *Tg(ins:il1b)* animals exhibited 2±0.4 L-plastin+ cells in the islet, this number was reduced to 0.5±0.4 cells in animals treated with 30 µM of wedelolactone (Fig. 6C; Movies 7 and 8). In order to assess whether this anti-inflammatory property is associated with a downregulation of NF-κB signalling in β-cells, we quantified GFP fluorescence intensity within the islet in the *Tg(NF-kB:EGFP)* reporter line after treatment with wedelolactone. Wedelolactone treatment (1-4 dpf) reduced the fluorescent intensity of GFP compared to controls, indicating that it suppresses the activation of NF-κB in the *Tg(ins:il1b)* model (Fig. 6C,D). These results indicate that wedelolactone treatment ameliorates islet immune-cell infiltration and β-cell inflammation.

**Fig. 7.**
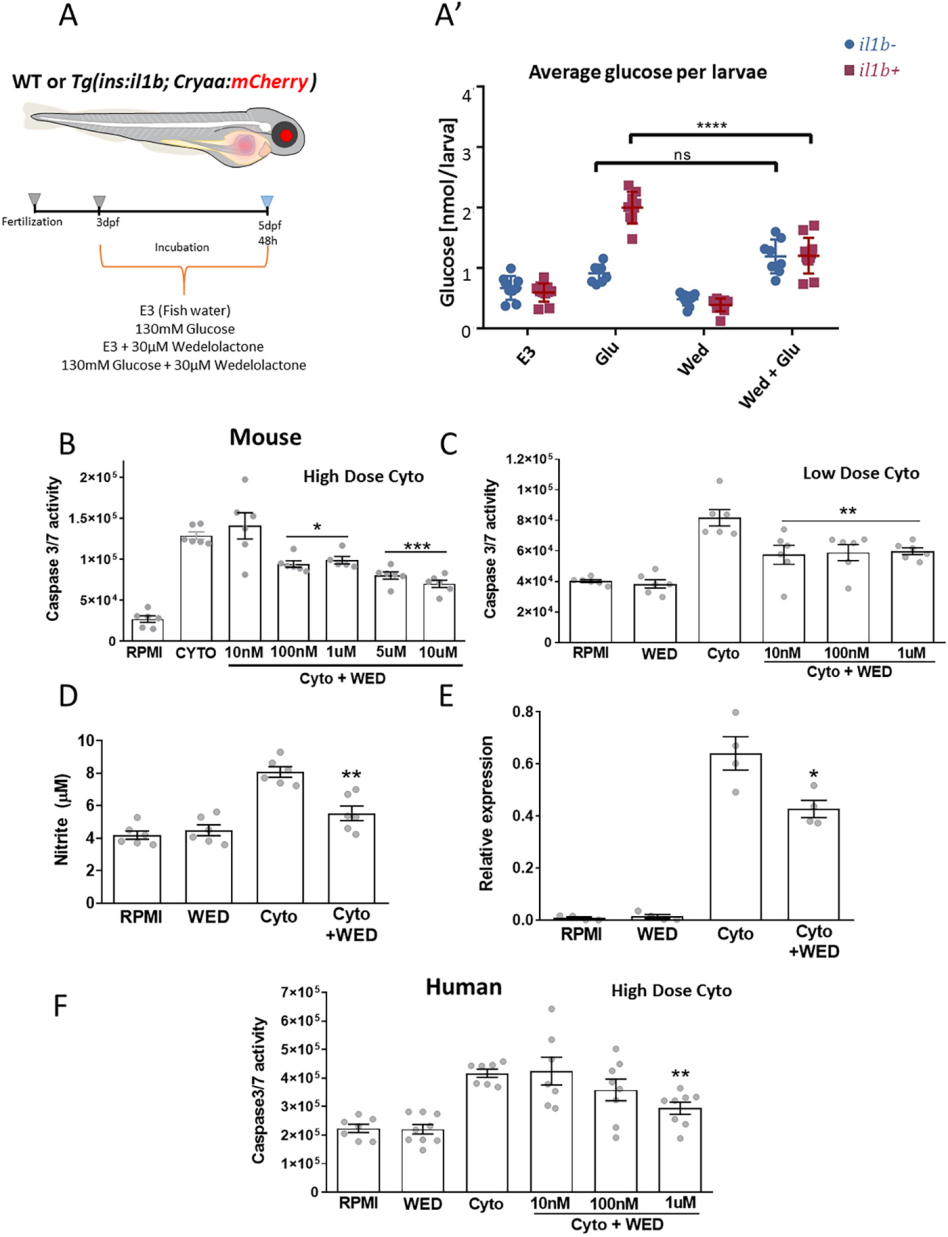
**Wedelolactone has antidiabetic properties *in vivo* and protects mouse and human islets from cytokine-mediated apoptosis.** (A) Larvae were treated with the natural product wedelolactone or DMSO during a glucose challenge. (A′) Plot showing average glucose values (nmol/larvae) following the treatment at 5 dpf. The *Tg(ins:il1b)* fish showed similar values as WT in normal fish water (E3). Upon glucose challenge, the *Tg(ins:il1b)* fish showed hyperglycemia. Wedelolactone treatment ameliorates the hyperglycemia in the *Tg(ins:il1b)* larvae following the glucose challenge. In all cases, 1% DMSO was used as a vehicle control. Two-way ANOVA with Sidak's multiple-comparison test; ****P≤0.0001; ns, not significant. Each data point represents a pool of ten larvae, mean±s.d. (B,C) Anti-apoptotic properties of wedelolactone in mouse islets upon exposure to high-dose (B) or low-dose (C) cytokine cocktails. (D) Wedelolactone decreased nitrite production following cytokine treatment of mouse islets. (E) Wedelolactone decreased iNOS expression in cytokine-treated mouse islets, as determined using the Greiss assay. (F) Anti-apoptotic properties of wedelolactone in human islets. In B-F, one-way ANOVA and Bonferroni's multiple-comparisons test. Data are presented as mean±s.e.m., *n*=6 biological replicates per treatment group. *P≤0.05, **P≤0.01, ***P≤0.005.

To examine whether wedelolactone can ameliorate the defect in glucose regulation, we repeated the glucose challenge in WT and *Tg(ins:il1b)* larvae in the presence of wedelolactone. Notably, wedelolactone treatment suppressed the ensuing hyperglycemia of *Tg(ins:il1b)* fish (Fig. 7A). Since wedelolactone can potentially inhibit the leukotriene production pathway as well, we tested a 5-LOX inhibitor, licofelone (Boileau et al., 2002), in the glucose-challenge assay. Treatment with licofelone suppressed the hyperglycemic phenotype of *Tg(ins:il1b)* larvae (Fig. S6). These data suggest that at least two pro-inflammatory pathways, NF-κB and leukotriene production, might be involved in the defective glucose regulation in *Tg(ins:il1b)* larvae.

### Wedelolactone protects mouse and human islets from cytokine-induced cell death

To test the predictive power of our zebrafish model to identify small molecules that could be used to treat diabetes, we determined the ability of wedelolactone to protect mouse and human β-cells from inflammation. To do this, we performed cytokine protection assays using cultured islets. To induce islet inflammation, a cocktail of recombinant inflammatory cytokines (TNF-α, 1000 U ml^−1^; IFN-γ, 1000 U ml^−1^; and IL-1β, 50 U ml^−1^) was applied to mouse islets in the presence of wedelolactone for 48 h. At the end of the experiment, we performed an assay that quantifies the levels of active caspase3/7, as a measure of islet-cell damage. Cytokine stimulation strongly enhanced the basal levels of caspase3/7 activity compared to controls (Fig. 7B). Importantly, wedelolactone treatment dose-dependently reduced caspase3/7 activity in this assay, indicating that it promotes β-cell survival (Fig. 7B). Similar protective results were observed under lower concentrations of the cytokines (Fig. 7C) and when wedelolactone was added to the cultured islets following exposure to cytokines (Fig. S7). The nitrite production pathway can be used as a surrogate marker of NF-κB activation. Wedelolactone treatment reduced nitrite levels in the supernatant, which were increased upon cytokine treatment (Fig. 7D). Since nitrite is produced via inducible nitric oxide synthase (iNOS), we also measured the relative expression levels of iNOS in islets treated with low doses of cytokines. Cytokine treatment increased iNOS expression, which was inhibited by wedelolactone (Fig. 7E). Finally, we assessed whether wedelolactone could also protect human islets from cytokines. Wedelolactone treatment strikingly reduced caspase3/7 activity in human islets exposed to the cytokine cocktail, indicating that its protective capacity is conserved in human cells (Fig. 7F). Altogether, wedelolactone prevents mouse and human β-cell death under inflammatory conditions and validates our zebrafish model for the identification of β-cell protective agents with translatable potential.

## DISCUSSION

Our study presents a new model of cytokine-driven β-cell dysfunction in zebrafish that is different from previous models, in which β-cells have been directly killed using cytotoxic means (Curado et al., 2008; Ninov et al., 2013; Pisharath et al., 2007). Such models are useful for understanding β-cell regeneration but cannot be used to study β-cell protection. This new model combined with our toolkit, which includes models allowing endogenous β-cell function to be monitored and the recruitment of innate immune cells to the islet to be evaluated, provides a powerful preclinical setting for evaluating the effects of candidate small molecules in β-cell protection. Furthermore, given the robust hyperglycemic phenotype, our model could be used to perform a screen for small molecules that improve glucose regulation, as a proxy for identifying agents that improve β-cell function during chronic inflammation. However, an important limitation of our model is the strong expression of *il1b* directly in the β-cells. Thus, there is a need for further refinement of the models of β-cell inflammation in zebrafish to establish a more physiological means to drive β-cell dysfunction.

Although much effort has been invested in strategies for β-cell regeneration via increasing proliferation or promoting *de novo* β-cell formation from other cells, fewer studies have explored the potential of β-cell protection. Pharmacological agents that reduce functional stress in β-cells, such as PPARγ agonists, GSK3β inhibitors and NMDA receptor antagonists, have been shown to protect human β-cells (Dalle et al., 2013; Gupta et al., 2010; Mussmann et al., 2007; Shimoda et al., 2011). Recently, the inhibition of a family of epigenetic modifiers, the Bromodomain and extra-terminal domain (BET), by the small molecule I-BET151 suppressed development of diabetes in the non-obese diabetic (NOD) mouse model (Fu et al., 2014) by promoting β-cell protection and regeneration. Moreover, an *in vitro* screen for drugs that inhibit β-cell dedifferentiation identified an Alk5 inhibitor as a potent small molecule capable of blocking the loss of β-cell identity (Blum et al., 2014). An important challenge will be translating small molecules with β-cell-protective properties to the clinic.

Translation could be facilitated through the repurposing of already clinically approved molecules for other diseases, as well as by the identification of natural products with therapeutic properties. In this regard, our study shows that wedelolactone, a natural plant product that is used in folk medicine to treat inflammatory conditions, exhibits anti-diabetic properties *in vivo* and produces β-cell protection in human islets. It will be important to conduct further tests in mouse models of diabetes to explore its efficacy before considering its translatability. In addition, it will be important to determine whether wedelolactone can arrest or reverse the loss of β-cell function in human β-cells.

The sequence of events that underlie β-cell loss in diabetes remains a subject of ongoing investigation. Formerly, it was assumed that β-cells succumb to chronic stress and undergo cell death. However, recent studies have challenged this concept, proposing that, under stressful conditions, β-cells can dedifferentiate, i.e. remain alive but no longer perform β-cell functions (Talchai et al., 2012). During the process of dedifferentiation, β-cells lose their own identity and instead express markers of other endocrine cells, such as α-cells. A recent paper also described the presence of β-cells with a silenced insulin promoter in long-standing T1DM patients (Kindt et al., 2017). Furthermore, cytokines were also shown to mediate human β-cell dedifferentiation *in vitro* (Nordmann et al., 2017). Our model of *in vivo* β-cell inflammation corroborates the idea that β-cell stress is associated with reduced functionality and perturbed β-cell identity. An important goal for the future will be to define the molecular mechanisms that allow β-cells to switch off their identity. Since the downregulation of β-cell identity might represent a natural defence mechanism used by β-cells to withstand conditions of metabolic stress and inflammation, it will be important to understand how to control this process if disease regression is to be achieved. In this regard, our *in vivo* GCaMP6s imaging suggests that the silencing of β-cell function can occur at the level of the inability of β-cells to mount a calcium response to glucose stimulation. Consistently, by transcriptomic analysis, we found that *kcnj11*, which encodes the components of the K-ATP channel, as well as the calcium channel genes *cacna1ha* and *cacng5a*, were downregulated in the *il1b*-expressing β-cells.

A notable feature of our model is the ability to conduct non-invasive imaging of the interactions between β-cells and immune cells. From the analysis of fixed samples, we obtained only a snapshot of these interactions, showing increased numbers of immune cells within the islet region. Importantly, the live-imaging aspect of our study was crucial in revealing the dynamic nature of these interactions. In fact, we found that the picture is far from static, with individual macrophages travelling in and out of the inflamed islet, while also showing significantly increased retention times. In the future, it will be informative to use our zebrafish model to study additional aspects of the crosstalk between β-cells and immune cells both under physiological conditions and during inflammation. In addition, our transcriptomic data generated from inflamed β-cells can be studied at the functional level to identify novel chemokines that orchestrate the dynamic interactions between immune cells and β-cells. Importantly, we show that the innate immune-cell infiltration in our model can be ameliorated using pharmacological means, suggesting that there is an active and tightly controlled dialogue between the immune cells and the β-cells during inflammation.

## MATERIALS AND METHODS

### Zebrafish husbandry

Zebrafish WT AB, WIK and TL were used in all the experiments. Zebrafish were raised in standard conditions at 28°C. Published transgenic lines used in this study were: *Tg(ins:GCaMP6s;cryaa:mCherry)* (Singh et al., 2017), *Tg(mpeg1:GAL4)* (Ellett et al., 2011), *Tg(UAS-Kaede)* (Davison et al., 2007), *Tg(ins:FUCCI-G1;cryaa:GFP)* (Ninov et al., 2013), *Tg(NF-kB:GFP)* (Kanther et al., 2011), *Tg(ins:nlsRenilla-mKO2)* (Singh et al., 2017), *TgBAC(tnfa:GFP)* (Marjoram et al., 2015) and *Tg(UAS:mCherry-NTR)* (Zhao et al., 2009). All experiments were carried out in strict compliance with European Union and German laws (Tierschutzgesetz), and with the approval of the TU Dresden and the Landesdirektion Sachsen (approval number: AZ 24D-9168, TV38/2015, A12/2016, A13/2016, TVV50/2017, TVV 45/2018 and all corresponding amendments). All live-imaging, glucose- and drug-treatment analyses were performed in larvae that did not exceed 5 dpf.

### Construction of *Tg(ins:il1b;cryaa:RFP)*

For the construction of *ins:il1b;cryaa:mCherry*, we used PCR amplification to remove the codons corresponding to amino acids 1-104 of the full-length *il1b* cDNA. A new ATG was inserted upstream of the coding region of the mature form of *il1b* and a FLAG tag was placed before the stop codon (C-terminus). During PCR, flanking 5′ *Eco*RI and 3′ *Pac*I sites were also introduced. A previously established plasmid backbone containing *ins:mAG-zGeminin;cryaa:mCherry* (Ninov et al., 2013) was digested with *Eco*RI/*Pac*I and the coding region of the truncated *il1b* gene was ligated using the *Eco*RI/*Pac*I sites. The entire construct was flanked with I-*Sce*I sites to facilitate transgenesis. Several founders were screened and one founder with Mendelian segregation was selected. This line was used in all further experiments.

### Glucose incubations

For the glucose challenge, larvae were incubated from 3 to 5 dpf in embryonic fish water (E3), 130 mM glucose or 130 mM mannitol. The larvae were kept at a density of 30 larvae per Petri dish in 50 ml volume of each solution. Following incubation, the larvae were rinsed with E3 and transferred in a new Petri dish containing fresh E3. After 5 min, ten larvae were collected in a 1.5 ml microcentrifuge tube, the E3 was removed and the fish were frozen in liquid nitrogen. The frozen fish were stored at −80°C until glucose measurement. The glucose-tolerance test was performed as previously described (Matsuda et al., 2017).

### Drug treatments in zebrafish

The following solutions were used: embryonic water (E3), 130 mM glucose, 30 µM wedelolactone, 30 µM wedelolactone+130 mM glucose. In all treatment groups, the solutions contained 1% DMSO. The larvae were incubated in a final volume of 50 ml with fish density of 30 larvae per dish. Compounds used were licofelone (5 μM) (Cayman no. 10007692; CAS no. 156897-06-2) and wedelolactone (15-30 μM) (Enzo BML-EI316-0001).

### Glucose measurements

The glucose levels in larvae were determined using the BioVision glucose assay kit. The larvae were stored at −80°C. Following thawing on ice, 250 µl of PBS were added. The larvae were then sonicated with an ultrasonic homogenizer (Bandelin SONOPLUS). The solutions were centrifuged at 13,000 ***g*** to pellet any insoluble residues. The glucose concentration was measured and calculated following the manufacturer's instructions (BioVision).

### Immunostaining and imaging

Larvae fixed in 4% PFA were permeabilized in 1% PBT (Triton X-100) and blocked for 2 h in 4% PBTB (BSA). Primary and secondary antibody stainings were performed overnight at 4°C. Primary antibodies were anti-insulin (guinea pig from DAKO, 1:200), anti-glucagon (mouse from Sigma, 1:500) and anti-L-plastin (rabbit, Biozol LS-C210139-250, 1:1000). A chicken polyclonal anti-GFP antibody (Abcam, ab13970) was used at 1:500 to detect *Tg*(*tnfa:GFP)* expression. Secondary antibodies were Alexa-Fluor-405 and Alexa-Fluor-488 anti-guinea pig (1:200); Alexa-Fluor-568 anti-rabbit (1:200); Alexa-Fluor-647 anti-mouse (1:200); and Alexa-Fluor-488 anti-chicken (1:200). Samples were mounted in Vectashield. Images were acquired using *z*-stacks on an LSM-780 Zeiss confocal microscope. For image analysis, the number of insulin- or glucagon-positive cells was counted using ImageJ. PowerPoint was used for adding arrows and labels to the images.

### Live imaging

Embryos were treated with 0.003% (200 µM) 1-phenyl 2-thiourea (PTU) to inhibit pigmentation from 24 hpf onwards. At 4.5 dpf, the larvae were anaesthetized using 0.4 g/l tricaine. The larvae were mounted in glass-bottom microwell dishes (MatTek corporation) using 1% low-melting agarose containing 0.4 g/l tricaine. After the agarose was solidified, the dishes were filled with embryonic fish water and 0.4 g/l tricaine.

### Live imaging and quantification of islets and macrophages

Live imaging of β-cells and macrophages was performed using the transgenic lines *Tg(ins:il1b);Tg(ins:mCherry-cdt1)* and *Tg(mpeg1:GAL4);Tg(UAS:Kaede)*, respectively. The larvae were mounted as described in ‘Live imaging’. Imaging was performed using an upright confocal microscope (ZEISS LSM 780) with an Achroplan 40×/N.A. 0.8 dipping lens. We acquired images of β-cells and macrophages simultaneously using the 488 nm and 561 nm laser lines. The videos were recorded every 5 min, for 10 h, with a *z*-step of 2 µm, covering 60 µm in total, and a resolution of 512×512 pixels. To quantify the duration that macrophages spent in the islet, we defined a region of interest (ROI) around the β-cells. We used the plugin ManualTracking in Fiji to track individual macrophages that entered the ROI. We further identified each macrophage in the confocal stack to make sure that the macrophage is in the plane of the islet. Finally, the time spent in the ROI was quantified.

### Live imaging of glucose-stimulated calcium influx in β-cells

Live imaging was performed in an inverted laser-scanning confocal system ZEISS LSM 780 inverted with a C-Apochromat 40×/N.A. 1.2 water correction lens using *Tg(ins:GCaMP6s);Tg(ins:FUCCI-G1)* larvae. We acquired the GCaMP6s and mCherry signals simultaneously using the 488 nm and 561 nm laser lines. The GCaMP6s signal was rendered in green and the nuclear signal in red. The videos were recorded every 15 s, with a *z*-thickness of 1.2 µm and a resolution of 512×512 pixels. Laser power was maintained as low as possible (<1.5%) to decrease phototoxicity.

The intra-cardiac glucose injection was performed as follows: a glass injection needle was made by pulling 3.5″ capillars (Drummond #3-000-203-G/X) with a micropipette puller (Sutter puller P-1000). The tip was cut and a volume of 5 nl was calibrated under a microscope. For injection, a pneumatic pico-pump was used (FemtoJet) with an injecting pressure of 500 hPa, and a compensation pressure of 0 hPa, delivered in a 1 s injection. The needle was carefully inserted into the heart in the agarose-mounted larva, with a micromanipulator (InjectMan N2). We injected 5 nl of 12.5 mM glucose solution in PBS. The injection of PBS alone did not elicit any response (*n*=3 islets).

For the quantification of the strength of response, we applied a maximum intensity projection to the confocal stacks. Using ImageJ, we delimited the area of the islet using the plugin ‘ROI manager'. In each frame, the GCaMP6s signal was extracted from the green channel as integrated fluorescence intensity. We divided the integrated intensity by the area of the ROI to estimate the fluorescent intensity (FI). The normalized FI values for the whole imaging time were obtained using the minimum and maximum values from the FI recorder during the imaging session using the following formula:

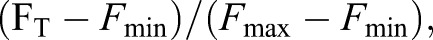
where F_T_ is the integrated fluorescence intensity at a given time; and *F*_max_ and *F*_min_ are the maximum and minimum values recorded during the live-imaging session, respectively.

For estimating the strength of the response, we calculated the fold change from the normalized FI before and after the glucose stimulation. For this, the FI was averaged from ten frames before the glucose stimulation, and ten frames after the glucose stimulation.

### Quantification of GFP-fluorescence intensity

To quantify *NF-kB*:GFP reporter expression, the larvae were fixed in 4% PFA and mounted in Vectashield. The skin covering the islet was removed using microdissection. Confocal stacks were acquired on a Zeiss LSM780. Maximum intensity projections were generated using the ImageJ software. The FI within an ROI was calculated as follows: by drawing the ROI around the boundaries of the islet, the integrated density was extracted. To calculate the mean fluorescence of the background, three ROIs in areas outside the islet were used to extract the mean grey value. Then the corrected total cell fluorescence (CTCF) was calculated: CTCF=Integrated density−(Area of selected islet×Mean fluorescence of background readings). To normalize across images, we divided the CTCF by the area of the islet.

### FACS of β-cells and gene expression analysis using mRNA sequencing

β-cells were isolated and sorted using FACS-Aria II (BD Biosciences). Briefly, the primary islet from *Tg(ins:il1b)* and *WT Tg(ins:nlsRenilla-mKO2)* fish at 3 mpf were manually dissected in ice-cold PBS. The islets were rinsed in PBS and then dissociated into single cells by digestion with TrypLE (ThermoFisher, 12563029), 0.1% Pluronic F-68 (ThermoFisher, 24040032) at 37°C, with shaking at 350 rpm for 50 min. After the dissociation, the digestion solution was neutralized with 10% FBS. The cells were then centrifuged for 10 min at 4°C at 500 ***g***. The supernatant was removed without disturbing the pellet. Cells were re-suspended carefully in 500 µl of HBSS (without Ca, Mg)+0.1% Pluronic F-68. To remove any undigested tissues, the cell solution was passed through a 30 µm cell filter (Miltenyi Biotec, 130-041-407). The total RNA was isolated using a Quick-RNA MicroPrep kit (R1050 Zymo Research), following the manufacturer's instructions. The sequencing was done on Illumina HiSeq2500 in 2×75 bp paired-end mode. The reads were mapped and aligned to the zebrafish genome GRCz10, using GSNAP from Ensembl gene annotation, version 87.

### Mouse and human islet experiments

Male mice were housed under temperature controlled (22±2°C) and 12 h light:12 h dark cycle conditions with *ad libitum* access to drinking water and standard rodent chow. Animal experiments were carried out in accordance with the British Home Office Animals Scientific Procedures Act 1986.

#### Islet isolation

Mouse islets were isolated from male CD1 mice aged 8-12 weeks (Charles River Laboratories, Margate, UK) by collagenase digestion (1 mg/ml; type XI; Sigma-Aldrich, Poole, UK) and separated on a histopaque gradient (Histopaque 1077; Sigma-Aldrich) (Franklin et al., 2018). Human islets were obtained from the pancreata of non-diabetic heart-beating donors with appropriate ethical approval (Huang et al., 2004). All islets were incubated overnight at 37°C (5% CO_2_) prior to experiments.

#### Islet apoptosis

Islets were cultured for 48 h with vehicle or wedelolactone at varying concentrations. During the last 20 h of culture, islets were exposed to either a high-dose cytokine cocktail (IL-1β, 0.2 ng/ml, TNF-α, 100 ng/ml, IFN-γ, 100 ng/ml) or a low-dose cocktail (IL-1β, 1 ng/ml; TNF-α, 5 ng/ml, IFN-γ, 5 ng/ml). Caspase3/7 activity was measured using the CaspaseGlo 3/7 kit as a measure of apoptosis (Promega, Southampton, UK) (Franklin et al., 2018). We measured iNOS expression and nitrite production as surrogate downstream markers of NF-κB activity. Islets were exposed to the low-dose paradigm. Total RNA was extracted from approximately 200 islets using an RNAeasy kit (Qiagen, Sussex, UK). Reverse transcription was performed using the High-capacity cDNA Reverse Transcription kit (Applied Biosystems, UK). Quantitative PCR amplification for iNOS expression was performed as previously described (Amisten et al., 2013). Nitrite production in the culture media was measured using the Measure-iT High Sensitivity Nitrite Assay Kit (Molecular Probes). Results shown are representative of data from three independent studies.

### Bioinformatics analysis

The differentially expressed genes were submitted to the PANTHER database (http://www.pantherdb.org/) and Gene Ontology (GO) analysis was performed. The 100 most upregulated or downregulated genes were used for the analysis. The terms GO-slim biological process was chosen. GO terms with a *P*-value less than 0.05 were further considered for analysis.

### Gene expression data

The mRNA sequencing data from this study are uploaded on Gene Expression Omnibus (GEO) under accession GSE123036.

### Statistical analysis

No statistical methods were used to predetermine sample size. The experiments were not blinded. Graphs were plotted using R. Statistical analysis was performed using Microsoft Excel and GraphPad. Values were compared using unpaired Student's *t*-test or ANOVA as indicated for each experiment. *P*-values <0.05 were considered statistically significant. Data are expressed as mean±s.d. unless otherwise specified.

## Supplementary Material

Supplementary information
