# Supplementary material for: Modelling pancreatic β-cell inflammation in zebrafish identifies the natural product wedelolactone for human islet protection
Source: Dis Model Mech. 2019 Jan 23;12(1):dmm036004. doi: 10.1242/dmm.036004 (PMC6361155; doi:10.1242/dmm.036004)
Supplement: Supplementary information [file dmm-12-036004-s1.pdf]

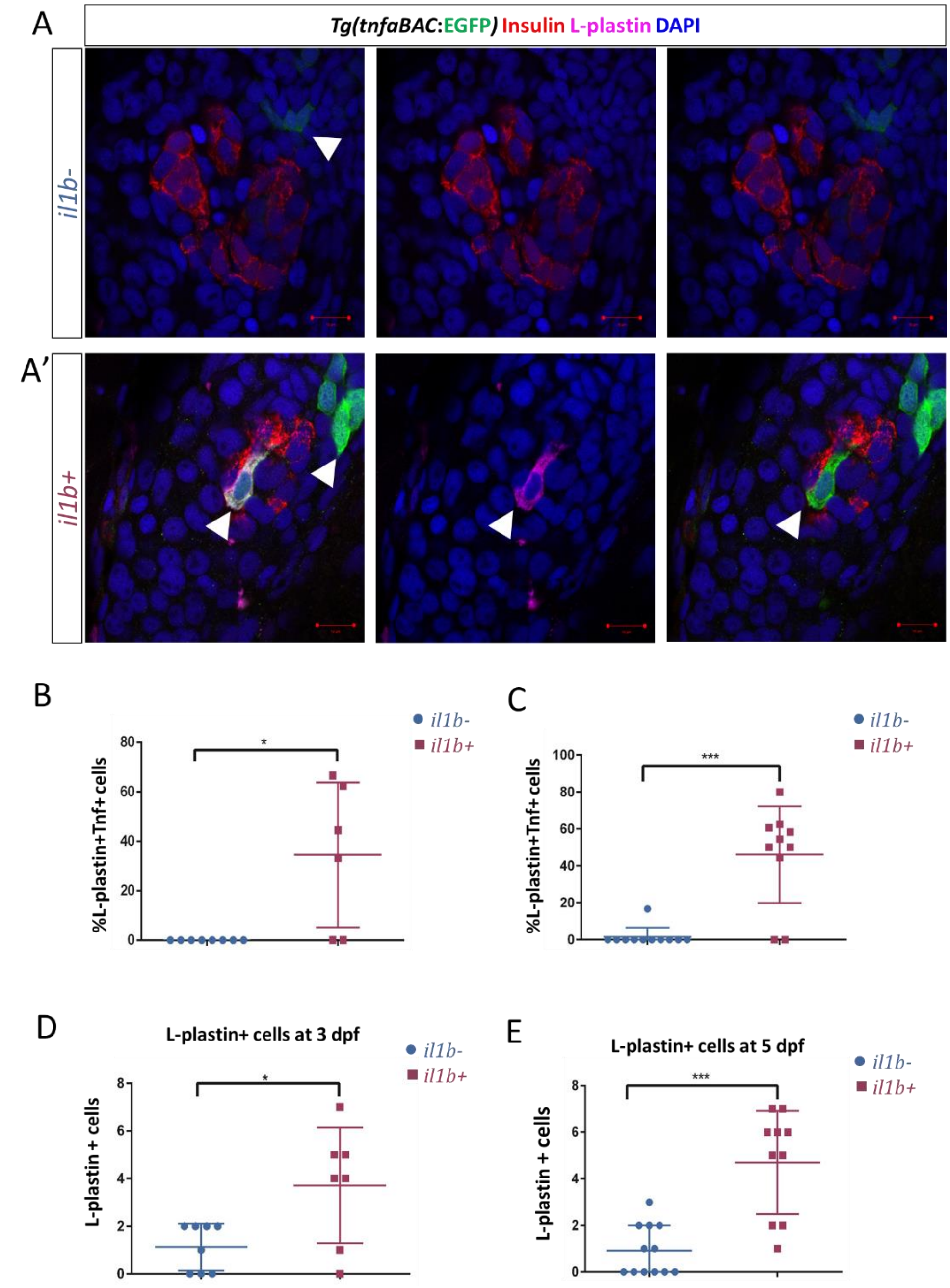

Figure S1. Leukocytes express *tnfa* in *Tg(ins:il1b)* larvae.
(A-A') Confocal slices (single plane) showing the primary islets of 3 dpf WT (A) and *Tg(ins:il1b)* larvae (A') in the background of a *Tg(tnfa:*GFP*)* transcriptional reporter. The arrow in A' shows an L-plastin-positive cell (magenta), which is also positive for *tnfa:*GFP (green). This cell is embedded in the islet. Some GFP-positive cells can be observed in the region corresponding to the extra-pancreatic duct in both WT and *Tg(ins:il1b)* larvae. These cells are L-plastin-negative. **(B-C)** Quantification of the proportion of GFP-positive cells among the L-plastin-positive cells in contact with the islet at 3 (B), unpaired two-tailed t-test with Welch's correction, *p-value ≤ 0.05, mean±SD and 5 dpf (C), unpaired two-tailed t-test with Welch's sorrection, ***p-value ≤ 0.001, mean±SD. **(D-E)** Quantification of the total number of L-plastin cells in contact with the islet at 3 (D), unpaired two-tailed t-test with Welch's correction, *p-value ≤ 0.05, mean±SD and 5 dpf (E), unpaired two-tailed t-test with Welch's correction, ***p-value ≤ 0.001, mean±SD . Scale bars in A-A'= 10 µm.

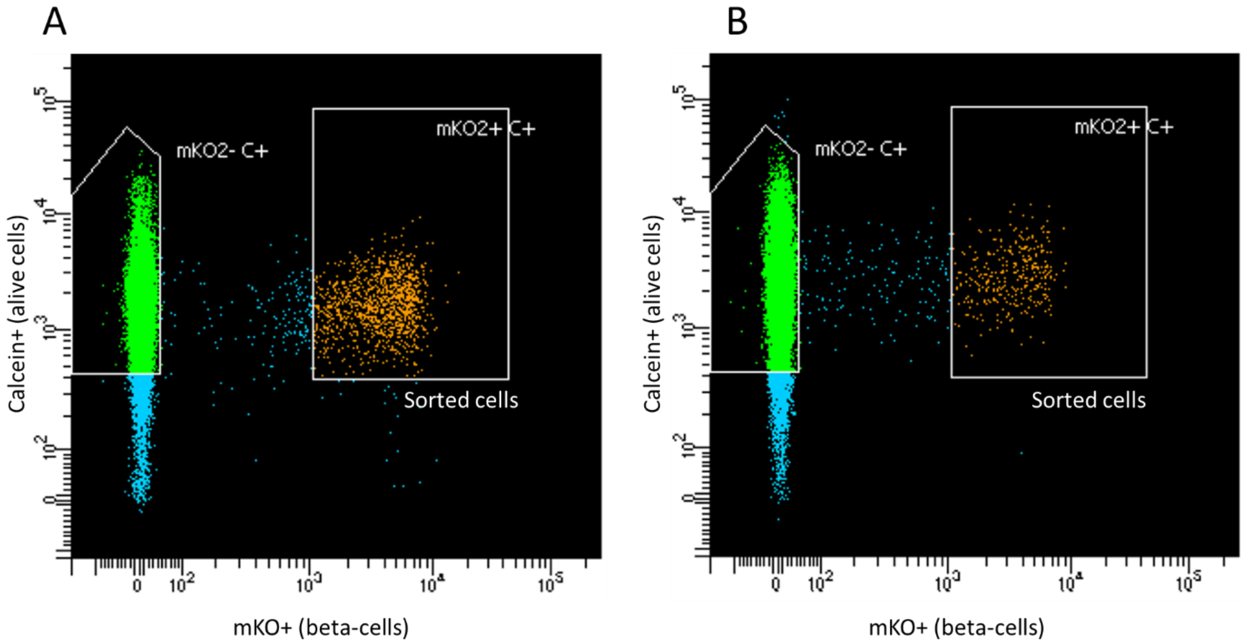

A’

Control			
Population	# events	% Parent	% Total
mKO+ Calcein +	1,190	4.3	2.4
mKO- Calcein +	24,414	87.8	48.8

B’

IL1B			
Population	# events	% Parent	% Total
mKO+ Calcein +	335	1.2	0.7
mKO- Calcein +	25,174	90.9	49.6

Figure S2. Fluorescence-activated cell sorting (FACS) of beta-cells.
(A-B) Representative plots showing the sorting of beta-cells from WT (A) and *Tg(ins:il1b)* (B) fish at 3 mpf. To mark beta-cells specifically, the *Tg(ins:nlsRenilla-mKO2)* reporter line was used, which expresses nuclear Kusabira-Orange under the insulin promoter.

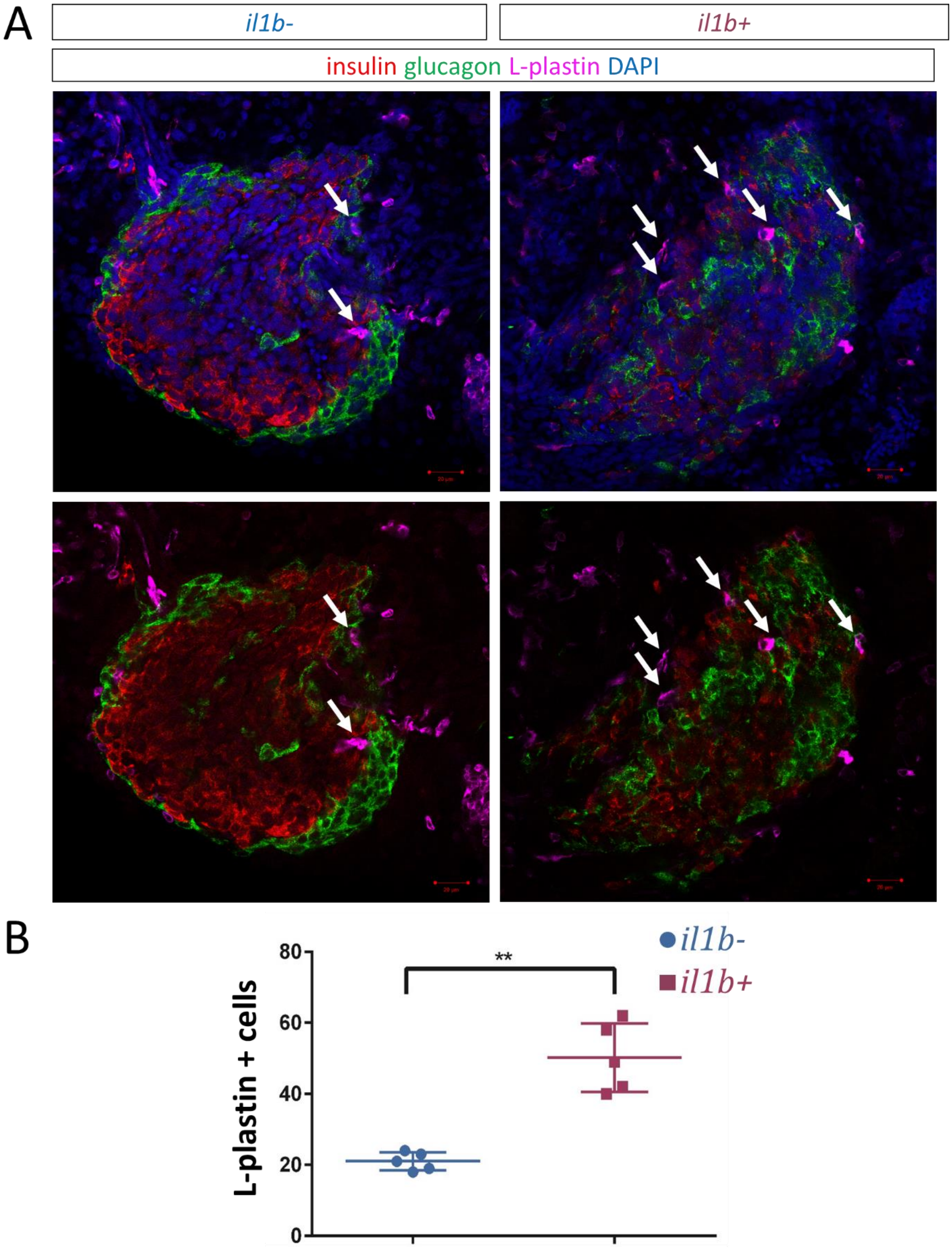

Figure S3. Persistent increase in immune cells within the islets of *Tg(ins:il1b)* fish at 30 dpf.

(A) Confocal slices (single plane) showing the primary islets in 30 dpf WT and *Tg(ins:il1b)* juveniles. Arrows point to L-plastin-positive cells. **(B)** Quantification of the number of L-plastin-positive cells per islet. *Tg(ins:il1b)* animals exhibit an increase in L-plastin-positive cells. Unpaired two-tailed t-test with Welch's correction. **p-value ≤ 0.01, mean±SD. Scale bars = 20 µm.

Disease Models & Mechanisms • Supplementary information

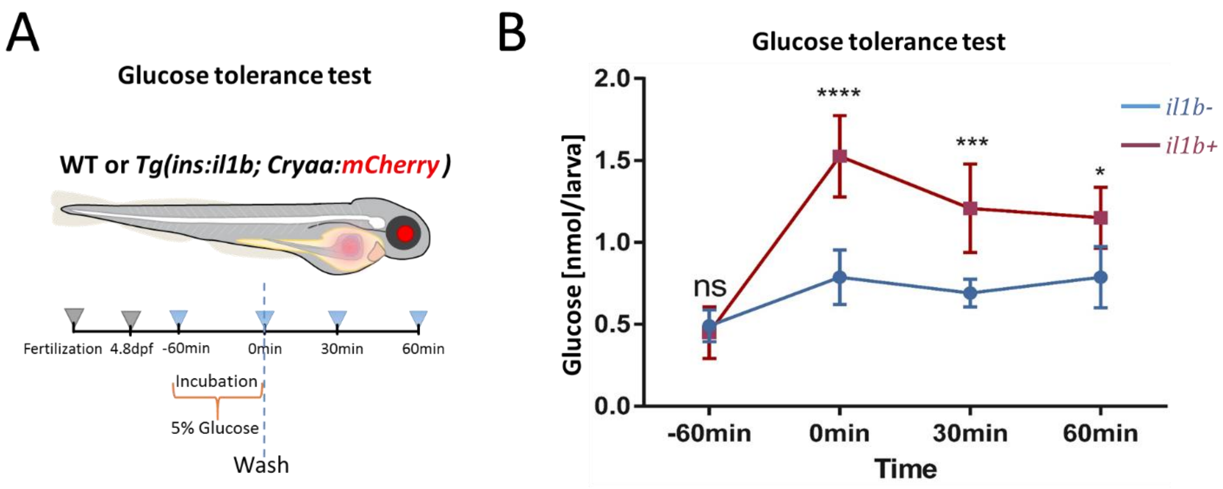

Figure S4. Impaired glucose tolerance in *Tg(ins:il1b)* larvae
(A) Schematic of the glucose-tolerance test. 4.8 dpf larvae were treated for 1h in 5% glucose. The glucose was washed away by rinsing the larvae several time and the larvae were placed in egg water (E3). Samples were collected before the glucose incubation (-60 min), and following the glucose stimulation (at 0, 30 and 60 min). **(B)** Graph showing the glucose levels in WT and *Tg(ins:il1b)* larvae at each time point from two independent experiments pooled together**.** Each data point represents a group of 10 larvae with n≥6 samples. Two way ANOVA with Sidak's multiple comparison test; ns: not significant, ****p-value ≤ 0.0001,***p-value≤0.001, *p-value≤0.05; mean±SD.

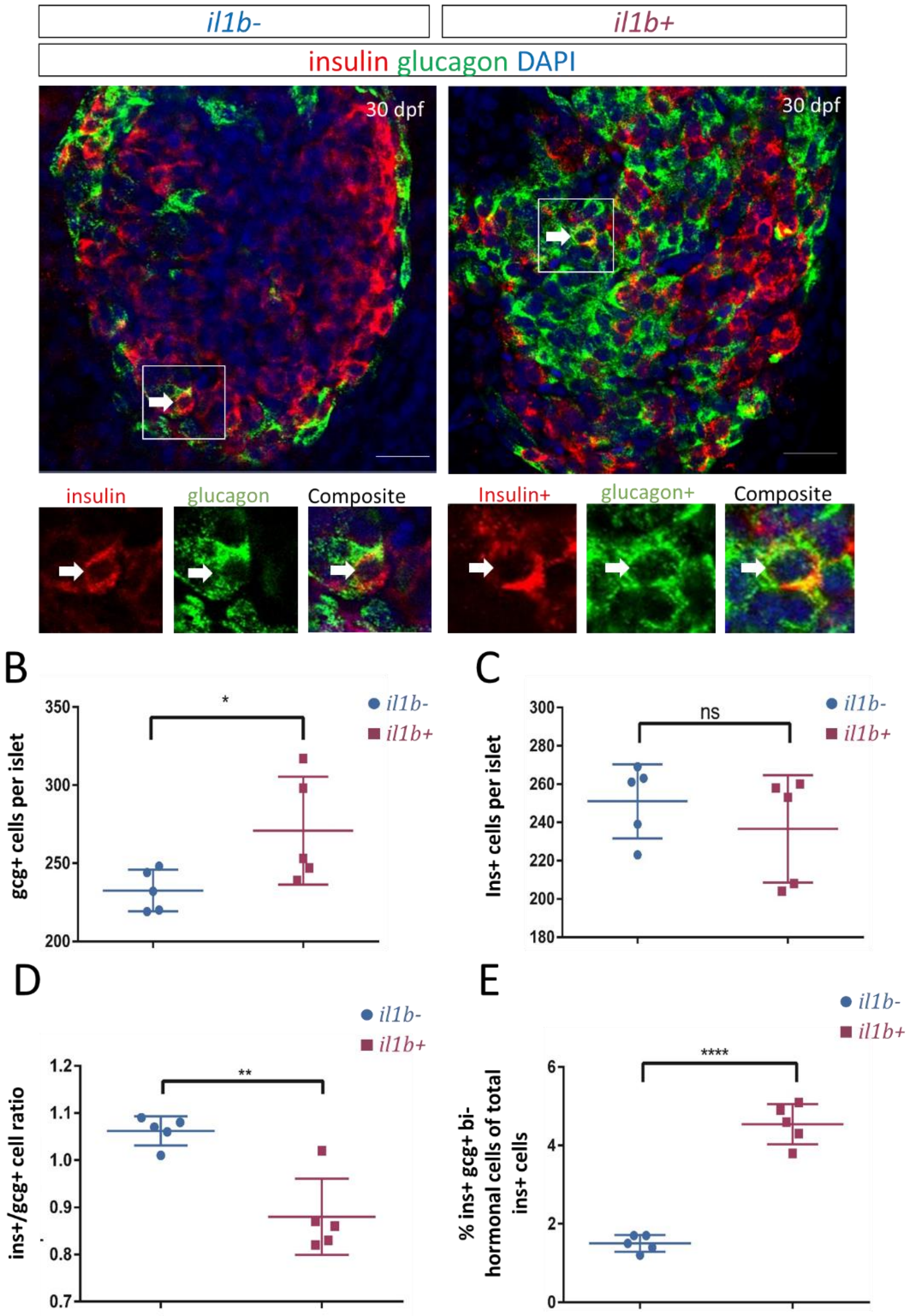

Figure S5. ***Tg(ins:il1b)*** **juveniles exhibit an increase in alpha-cells and higher proportion of insulin-positive cells that co-express glucagon.**

(A) Confocal slices (single plane) showing the primary islets in 30 dpf WT and *Tg(ins:il1b)* juveniles. The higher-magnification insets show insulin/glucagon double-positive cells corresponding to the outlined region in the top panels (arrows). **(B)** Quantification of the number of glucagon-positive cells per islet. *Tg(ins:il1b)* animals exhibit an increase in glucagon-positive cells. Unpaired two-tailed t-test with Welch's correction; *p-value ≤ 0.05, mean±SD. **(C)** Quantification of the number of insulin-positive cells per islet. Unpaired two-tailed t-test with Welch's correction, ns: not significant. mean±SD. **(D)** Quantification of the ratio of insulin-to-glucagon-positive cells per islet. Unpaired two-tailed t-test with Welch's correction; **p-value ≤ 0.01, mean±SD. **(E)** Quantification of portion of insulin positive cells per that co-express glucagon over the total number of insulin-positive cells per islet. Unpaired two-tailed t-test with Welch's correction; ****p-value ≤ 0.0001, mean±SD. Scale barsin A = 20 µm.

Disease Models & Mechanisms • Supplementary information

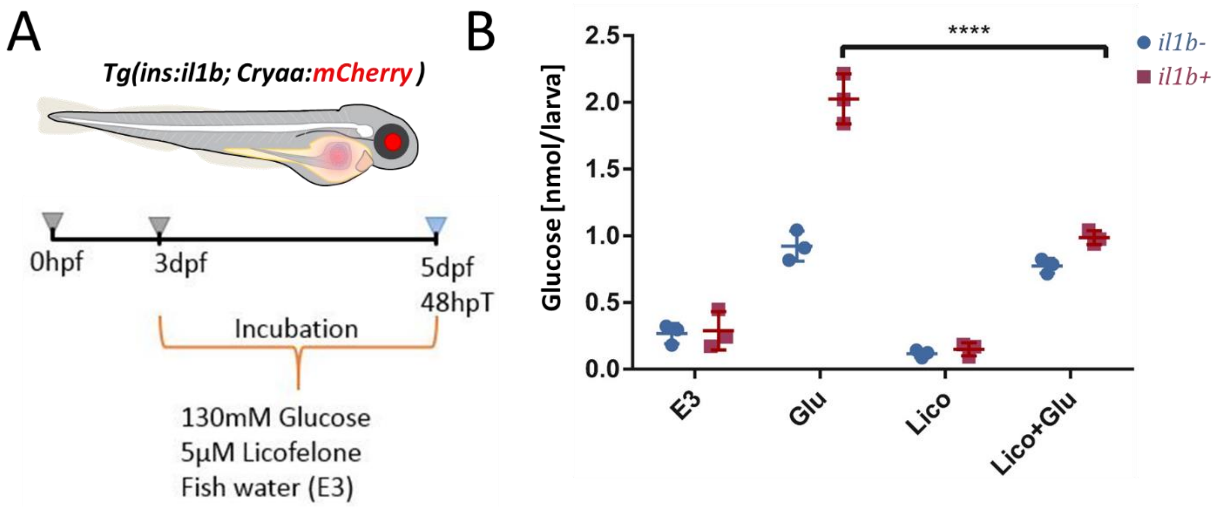

Figure S6. The 5-LOX inhibitor Licofelone ameliorates the hyperglycemia in *Tg(ins:il1b)* larvae.
(A) Schematic of the experimental setup. Larvae were treated with 5µM Licofelone or DMSO during a glucose challenge from 3-5 dpf. Glucose was measured at 5 dpf. **(B)** Plot showing average glucose values [nmol/larvae] following the treatment. The *Tg(ins:il1b)* fish showed similar values as WT in normal fish water (E3). Upon glucose challenge, the *Tg(ins:il1b)* fish showed hyperglycemia. Licofelone-treatment ameliorated the hyperglycemia in the *Tg(ins:il1b)* larvae following the glucose-challenge. In all cases, 1% DMSO was used as a vehicle control. Two way ANOVA with Sidak's multiple comparison test; ****p-value ≤ 0.0001. Each data point represents a pool of 10 larvae, mean±SD.

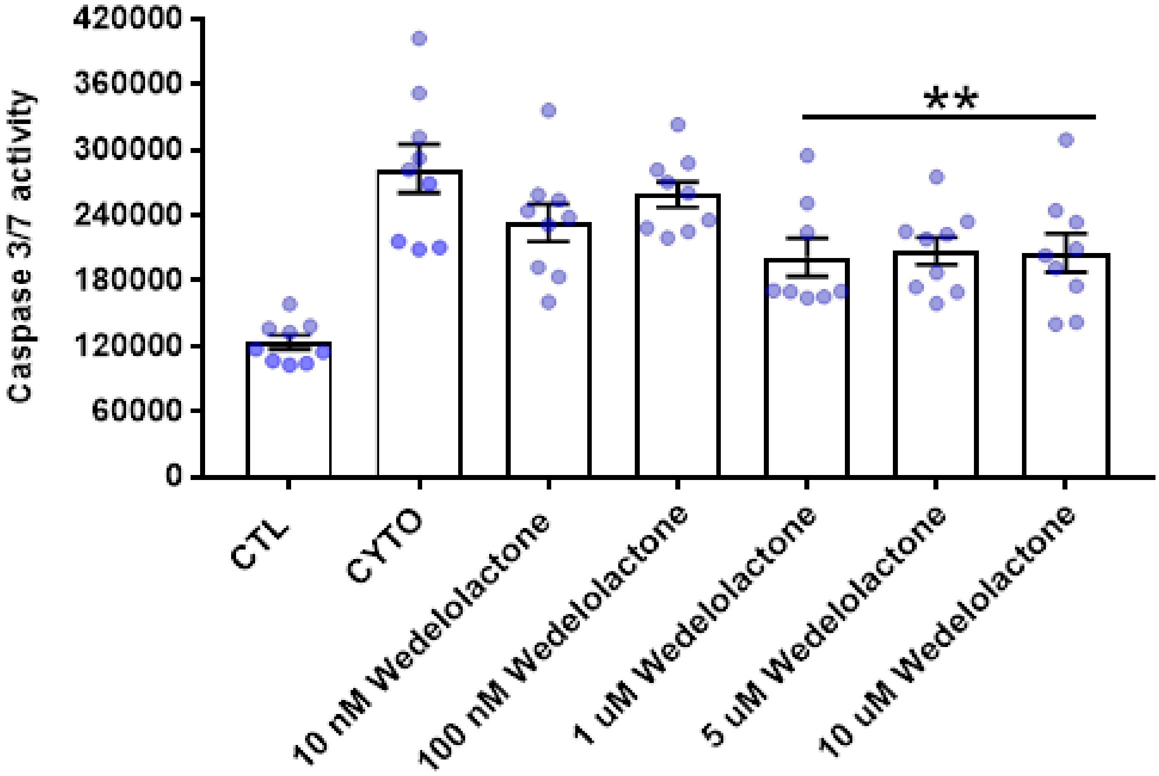

Figure S7. Wedelolactone inhibits cytokine-induced islet-cell apoptosis without pre-treatment.
Mouse islets were incubated with increasing concentrations of Wedelolactone (10nM-10 uM) , in the presence of a cytokine cocktail for 20 hours. Apoptosis was assessed by Caspase 3/7 activity. n = 9 biological replicates per treatment group. **p≤0.01 by one way Annova with Dunnett's post hoc analysis.

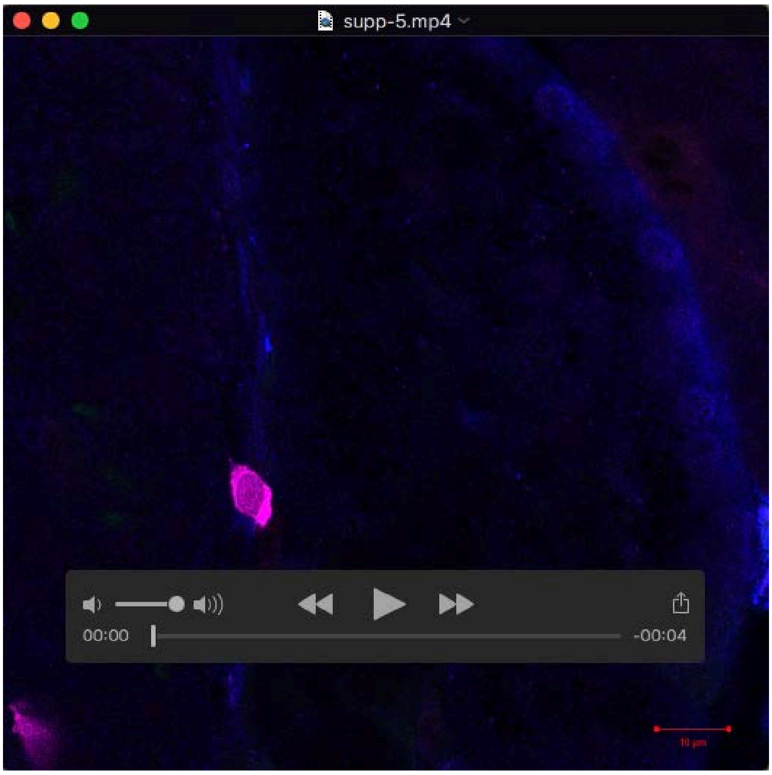

Movie 1. A confocal stack showing beta-cells (blue), Nf-kB activity (green) and L-plastin-positive cells in WT larvae.

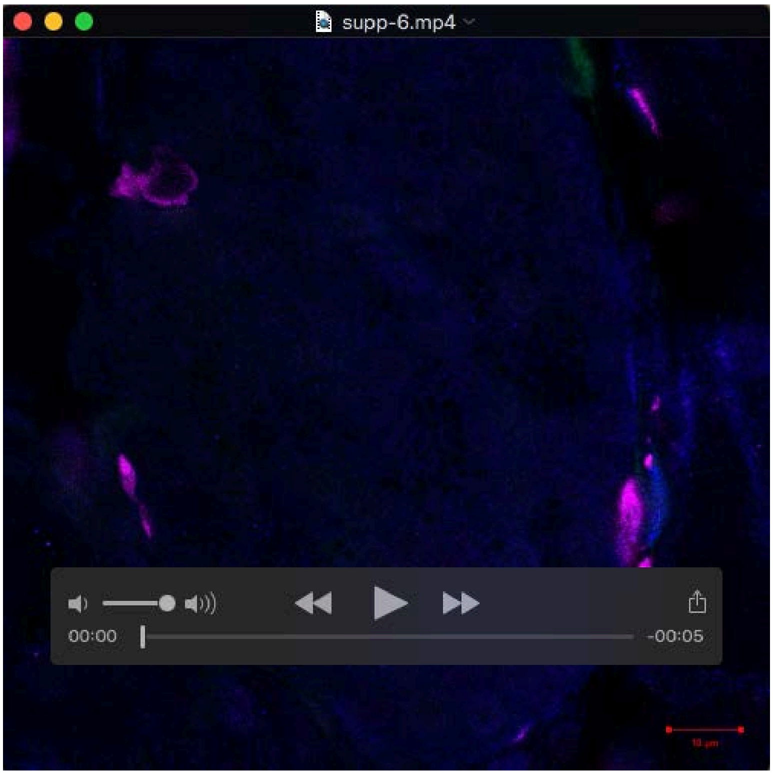

Movie 2. A confocal stack showing beta-cells (blue), Nf-kB activity (green) and L-plastin-positive cells in *Tg(ins:Il1b)* larvae.

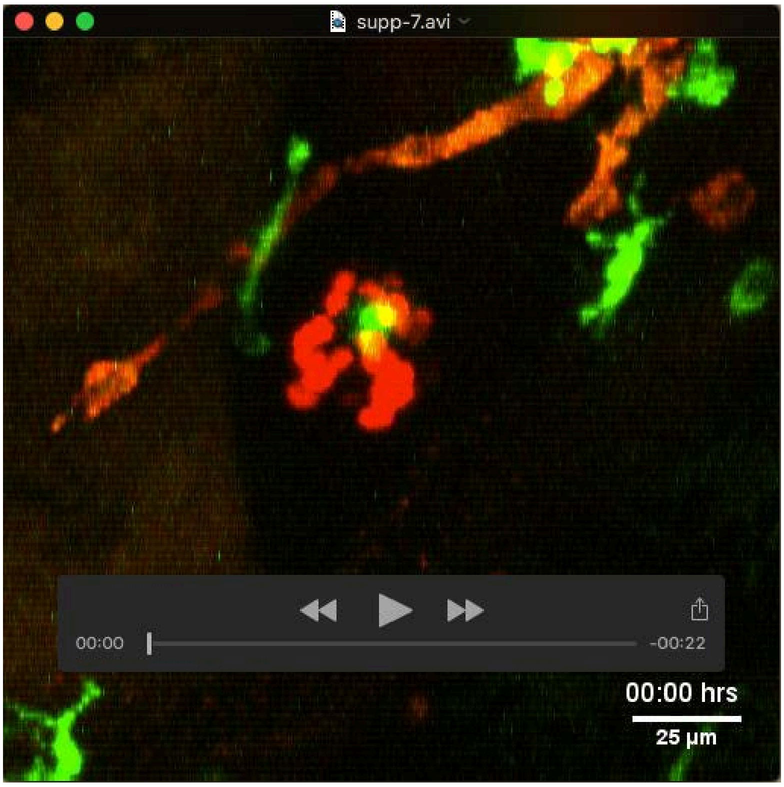

Movie 3. *In vivo* live imaging of macrophages (green) and beta-cells (red) in control larvae.

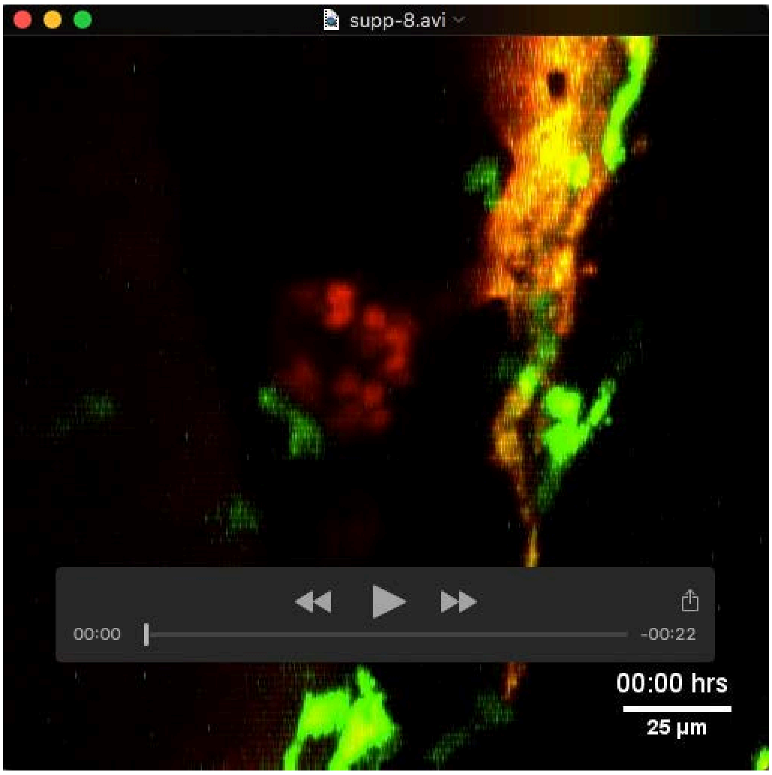

Movie 4. *In vivo* live imaging of macrophages (green) and beta-cells (red) in *Tg(ins:Il1b)* larvae.

Disease Models & Mechanisms • Supplementary information

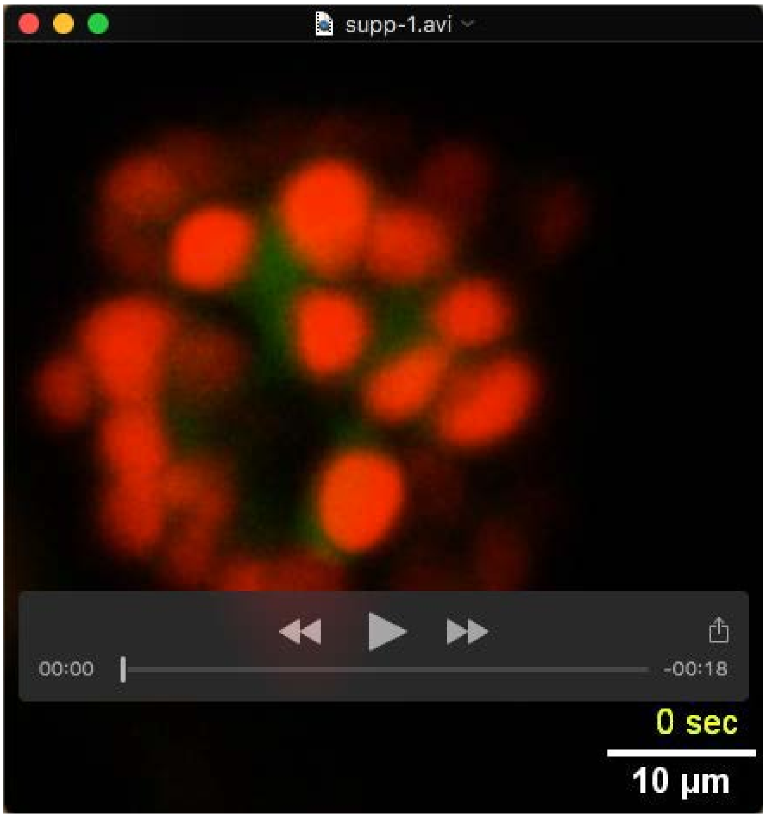

Movie 5. *In vivo* live imaging of glucose-stimulated calcium influx in beta-cells in control larvae

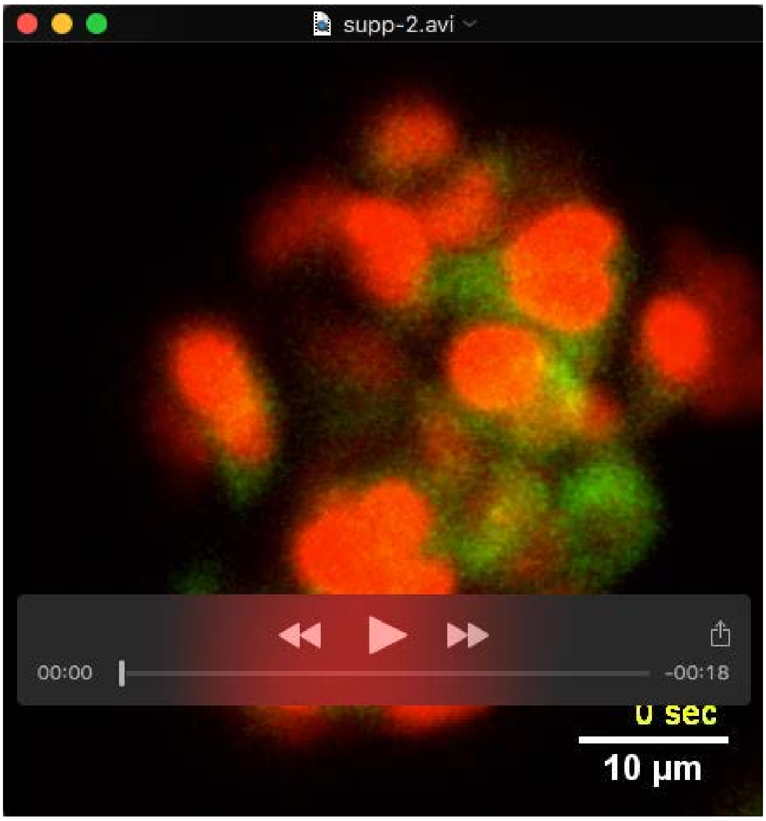

Movie 6. *In vivo* live imaging of glucose-stimulated calcium influx in beta-cells in *Tg(ins:Il1b)* larvae.

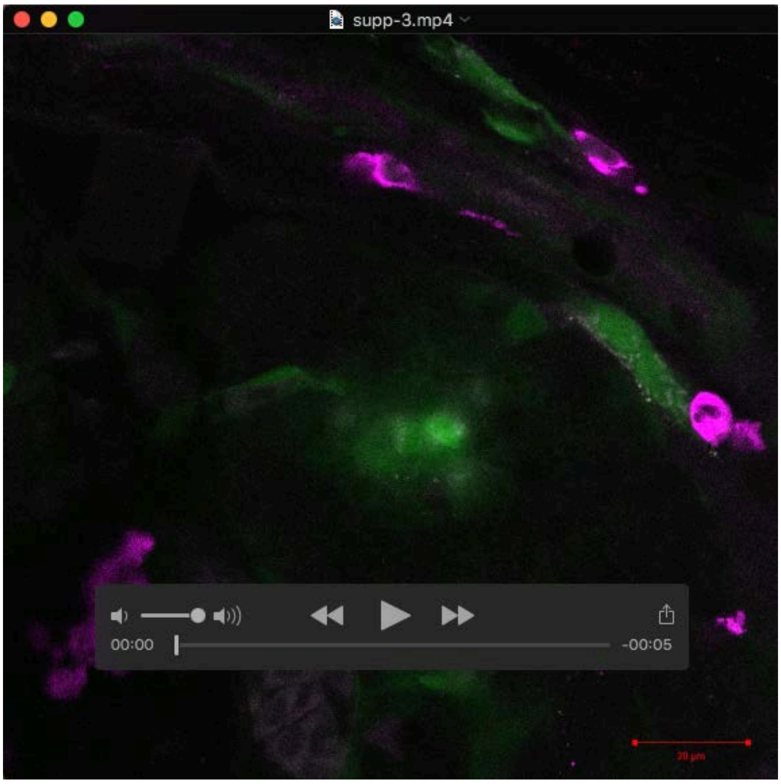

Movie 7. A confocal stack showing beta-cells (white), Nf-kB activity (green) and L-plastin-positive cells (magenta) in *Tg(ins:Il1b)* larvae treated with DMSO.

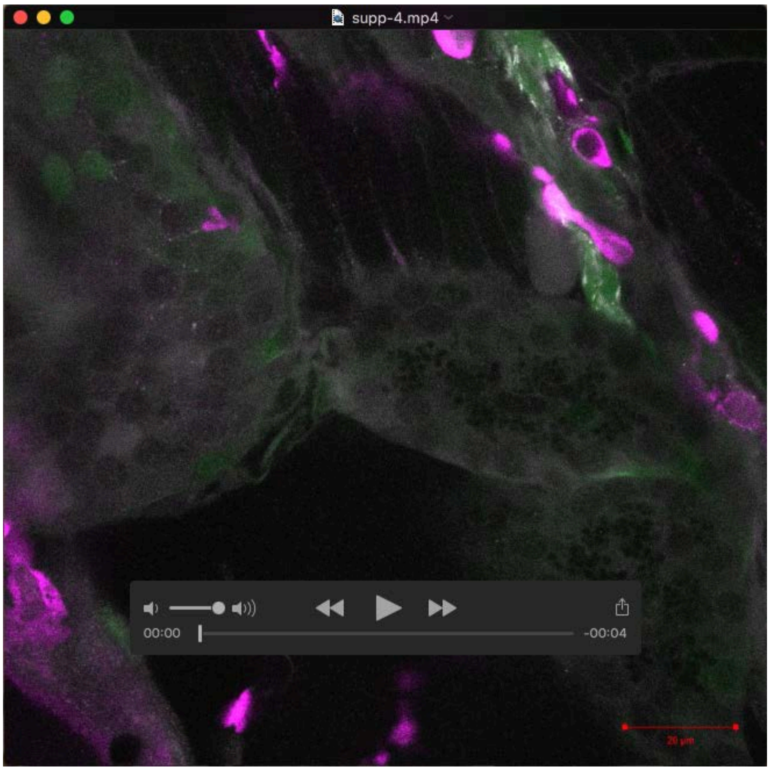

Movie 8. A confocal stack showing beta-cells (white), Nf-kB activity (green) and L-plastin-positive cells (magenta) in *Tg(ins:Il1b)* larvae treated with Wedelolactone.
